# Comprehensive bioinformatics analysis reveals prognostic significance and immunological roles of WNT gene family in breast cancer

**DOI:** 10.1038/s41598-025-13315-6

**Published:** 2025-10-03

**Authors:** Fatema Tuj Johora Fariha, Muntasim Fuad, Chandra Shekhar Saha, Sajjad Hossen, Md. Jubayer Hossain

**Affiliations:** 1 Center for Health Innovation, Research, Action, and Learning-Bangladesh (CHIRAL Bangladesh), 100, Shukrabad, Mirpur Road, Dhanmondi, 1207 Dhaka, Bangladesh; 2 Big Bioinformatics Lab, Center for Health Innovation, Research, Action, and Learning-Bangladesh (CHIRAL Bangladesh), 100, Shukrabad, Mirpur Road, Dhanmondi, 1207 Dhaka, Bangladesh; 3 Department of Microbiology, Jagannath University, Dhaka, Bangladesh

**Keywords:** WNTs, Breast cancer, Prognostic biomarkers, Expression patterns, Immunology, Cancer treatment, Bioinformatics, Cancer, Cell biology, Computational biology and bioinformatics, Genetics, Immunology, Systems biology, Biomarkers, Diseases, Health care, Medical research, Oncology

## Abstract

Breast cancer (BRCA) is one of the most commonly diagnosed cancers and the leading cause of cancer-related deaths among women worldwide. Previous studies have shown that the WNT (wingless type) gene family plays a role in the development of various cancers. However, comprehensive analysis of WNTs in BRCA remains largely unexplored. In this study, we examined the expression patterns, clinical relevance, and survival outcomes associated with the WNT family and identified key prognostic WNTs. We further investigated genetic alterations, DNA methylation, and drug sensitivity using cBioPortal, SMART, and GSCA databases. Data from GEO and DepMap were used for validation. Our findings revealed that while WNT2 and WNT7B were significantly upregulated and WNT11 was downregulated, WNT2 paradoxically correlated with favorable prognosis despite its oncogenic overexpression. Amplification was the most common type of alteration among the key WNTs selected for analysis and was correlated with immune infiltration and immunotherapy-related biomarkers. Functional enrichment analysis uncovered a novel connection between WNT signaling and neurodegenerative pathways. Furthermore, co-expression and drug sensitivity analyses revealed that WNTs influence the efficacy of multiple anti-cancer agents, offering potential targets for precision oncology. This study provides novel insights into the multifaceted role of WNT genes in BRCA, identifying them as promising prognostic indicators and immunotherapeutic targets that warrant further clinical exploration.

**Supplementary Information:**

The online version contains supplementary material available at 10.1038/s41598-025-13315-6.

## Introduction

Breast cancer is one of the most prevalent malignancies and the primary cause of cancer-related mortality in women globally^1,2^. In 2022, approximately 2.3 million new cases (11.6% of all cancer cases) and 666,000 deaths (6.9% of all cancer deaths) were documented in women across 157 countries for incidence and 112 countries for mortality, projected to rise to nearly 3 million by 2040^3,4^. The incidence of breast cancer is expected to increase in East and South Asian countries, with age-standardized death rates increasing by 7.0% and 35% from 1990 to 2030, respectively^5^. The heterogeneous nature of breast cancer, characterized by diverse genetic, epigenetic, histopathological, and clinical features, as well as frequent resistance to various therapies and the development of recurrence and metastasis, poses significant challenges in clinical management^6^. Therefore, understanding the molecular mechanisms underlying breast carcinogenesis is crucial to develop more effective and personalized treatment approaches.

The human genome contains 19 WNT genes that encode highly conserved secreted glycoproteins^7^. These proteins are hydrophobic, notoriously insoluble, rich in cysteine, with molecular weights ranging from 39 to 46 kDa, and consist of 350–400 amino acids^8^. WNT proteins are secreted via the endoplasmic reticulum (ER) and Golgi apparatus and play diverse roles in various cellular and biological processes, including cell proliferation, differentiation, polarity, migration, apoptosis, survival, embryonic development, stem cell maintenance, and tissue homeostasis^9^. WNT signaling is categorized into two main pathways: (1) the canonical (β-catenin-dependent) pathway and (2) the non-canonical (β-catenin-independent) pathway^10^. In the canonical pathway, WNT binding stabilizes β-catenin, preventing its degradation and allowing its nuclear translocation, where it regulates the transcription of genes involved in cell proliferation and survival^11^. Non-canonical pathways independent of β-catenin are involved in cell movement and polarity. WNT signaling is initiated when WNT binds to Frizzled (Fz) receptors along with low-density lipoprotein (LDL) receptor-related proteins (LRP) on the cell surface^12^. This binding triggers a cascade involving various intracellular proteins, such as Dishevelled (Dsh), glycogen synthase kinase-3β (GSK-3β), Axin, Adenomatous Polyposis Coli (APC), and β-catenin^13^.

Abnormal WNT activity frequently contributes to cancer progression, metastasis, and treatment resistance, particularly in colorectal, breast, and liver cancers^14,15^. Both genetic alterations and epigenetic modifications, such as promoter hypermethylation of WNT inhibitors in the WNT family, have been associated with human malignancies^16^. WNT signaling promotes cell division by activating transcription factors of the TCF/LEF family of target genes, including c-MYC and cyclin D1, which are essential regulators of cell cycle progression. Additionally, WNT signaling can inhibit cell cycle arrest by modulating CDK inhibitors, such as p21 and p27^17^. When WNT signaling is dysregulated, often due to mutations in its components, such as APC, β-catenin can also suppress tumor suppressor pathways, leading to unchecked cell proliferation^18^. Moreover, WNT signaling interacts with several other pathways, including PI3K/AKT, Hippo, Notch, MAPK/ERK, and p53, which are implicated in tumorigenesis^19^. Beyond cancer, aberrant expression of WNTs has been linked to various diseases such as osteoporosis and degenerative disorders^20,21^.

Thus, identifying abnormal WNT gene expression could serve as a biomarker for early tumor detection, and targeting WNT signaling may offer a novel and promising approach for cancer treatment. In this study, we evaluated the expression of WNT in clinical samples from Breast with Carcinoma (BRCA) patients using the UCSC XENA and GEPIA Web portals. Here, we report that the expression of key WNTs is significantly deregulated in BRCA. Next, we proceeded with prognostic significance, genetic alterations, epigenetic modifications, ratio of immune cell infiltration and immune therapy-related genes, enrichment analysis, and responsiveness of key WNTs to drugs (Table 1). This analysis demonstrated the potential molecular mechanism of WNTs in BRCA progression and highlighted their roles as prognostic biomarkers. These key WNTs may be used for early detection, targeted therapy, or personalized medicine in the treatment of patients with BRCA.

**Table 1:** List of the web-tools, databases, software and R packages used in the study.

Web tools/Software/ *R* packages	Data type	Analysis type	Database	URL
UCSC XENA	Gene expression	Heatmap showing the expression of genes among normal breast tissue, solid normal tissue surrounding the tumor, and primary tumor	TCGA GTEx	https://xenabrowser.net/
GEPIA2	Gene expression	BRCA vs. normal breast tissue analysis	TCGA GTEx	http://gepia2.cancer-pku.cn/
UALCAN	Gene expression	Gene expression based on clinicopathological characteristics of BRCA	TCGA	http://ualcan.path.uab.edu/index.html
TIMER2.0	Gene expression levels across various pan-cancers	Gene expression levels across different cancer types	TCGA	http://timer.cistrome.org/
Kaplan-Meier Plotter	Gene expression & patient prognosis data	Survival Analysis	TCGA	https://kmplot.com/analysis/
GEOquery (version 2.74.0) R package	Expression profiling by array	Retrieving data from NCBI GEO	GEO	https://seandavi.github.io/GEOquery/
DESeq2 (v1.46.0) R Package	Expression profiling by array	Differential gene expression analysis	GEO	https://bioconductor.org/packages/DESeq2
EnhancedVolcano R package (v1.24.0)	Differential gene expression data	Visualization of differential expression results	GEO	https://bioconductor.org/packages/EnhancedVolcano
DepMap	Cancer cell line analysis	Cell lines with gene effect scores	Achilles and Sanger’s SCORE projects	https://depmap.org/
TCGAplot R package (v8.0.0)	Gene expression	Gene-gene correlation analysis	TCGA	https://github.com/tjhwangxiong/TCGAplot
ggplot2 R Package (v3.5.1)	Breast cancer cell lines and gene effect score data	Barplot visualizing cancer line analysis results	DepMap	https://ggplot2.tidyverse.org/
SMART	Promoter DNA methylation	DNA methylation analysis	TCGA	http://www.bioinfo-zs.com/smartapp/
cBioPortal	Genetic alteration	Frequency of mutation, amplification, deep deletion and multiple alterations across various BRCA studies	TCGA	https://www.cbioportal.org/
AlphaFold	Protein structure predictions	Predicting protein 3D structures from amino acid sequences	AlphaFold Protein Structure Database	https://alphafold.com/
TCGAplot R package (v8.0.0)	Immune infiltration correlation and immune-related genes correlation	Gene expression Immune cell ratio, immune score, chemokines, chemokine receptors, immune checkpoint genes (ICGs), immune inhibitors, and immune stimulators correlation analysis	TCGA	https://github.com/tjhwangxiong/TCGAplot
ggVennDiagram (v1.5.3) R package	Positively correlated immune-related genes	Venn diagram visualizing common Positively correlated immune-related genes	Correlation analysis results	https://gaospecial.github.io/ggVennDiagram/
STRING	Protein-protein interaction	Protein-protein interaction network visualization	Proteins and their interactions	https://string-db.org/
clusterProfiler (v4.14.4) R package	Genomic data (gene expression, gene sets, pathways)	Functional enrichment analysis	KEGG, Reactome, GO	https://yulab-smu.top/biomedical-knowledge-mining-book/
Gene Set Cancer Analysis (GSCA)	Drug Sensitivity	Correlation between drug sensitivity and mRNA expression	GDSC, CTRP	https://guolab.wchscu.cn/GSCA/#/drug
ggpubr (v0.6.0) R Package	Correlation between drug sensitivity and mRNA expression results	Visualization of Drug Sensitivity Analysis	GDSC, CTRP	https://rpkgs.datanovia.com/ggpubr/

## Methods

### Analysis of the expression patterns of WNTs in breast cancer

We used UCSC XENA (https://xenabrowser.net/), a web-based, high-performance interaction visualization, exploration, and analysis tool for multi-omics cancer data, to analyze the expression patterns of WNTs in breast cancer^22^. We generated a heatmap comparing the expression of WNTs among normal breast tissue, solid normal tissue surrounding the tumor, and primary tumor by following these steps: A. Select a study to explore: “TCGA target GTEx” as study, B. Select Data Type: Genomic, input all 19 genes from WNT family in ‘Add gene or Position’ and selected “Gene Expression” under “Basic” as Dataset, C. Select Data Type: Phenotype, and selected “Basic” under “primary_site,” then we typed “breast” to select samples and used “Keep Samples” to filter breast as the only primary site. We retrieved Gene Symbol, Gene ID, log2(Fold Change), and adjp value using “Differential Genes” from GEPIA2^23^. We used the “Expression DIY” module of GEPIA2 to obtain box plots of gene expression in BRCA and normal breast tissues. An adjusted P-value of < 0.05 was considered significant for both analyses.

### WNTs expression based on clinicopathological characteristics of breast cancer

UALCAN (http://ualcan.path.uab.edu/) is a comprehensive, facilitative, interaction-based online resource for analyzing cancer omics data and clinical tumor information from TCGA^24^. Using the UALCAN platform, we examined the expression levels of WNT2, WNT7B, and WNT11 in breast cancer across diverse clinical parameters. The parameters included cancer stage, tumor subtype, patient age, and menopausal status. A P-value of < 0,05 was considered statistically significant at a comparison.

### Pan-cancer view of WNTs

The TIMER2.0 (http://timer.cistrome.org/) database is an excellent resource for systematically analyzing the connections between gene expression and tumor characteristics in TCGA^25^. We used the TIMER database to assess the expression levels of WNT2, WNT7B, and WNT11 in various pan-cancers. We used the “Gene_DE” module to compare the expression profiles of these genes across different cancer types and contrasting tumor and normal tissue samples. Statistical significance determined using the Wilcoxon test is indicated by asterisks: * *p* < 0.05, ** *p* < 0.01, and *** *p* < 0.001.

### Survival prognosis analysis across the BRCA cohort

To evaluate the prognostic significance of the WNT2, WNT7B, and WNT11 genes in BRCA, we used the Kaplan-Meier Plotter (https://kmplot.com/analysis/), which is mainly based on Affymetrix microarray information from the TCGA database^26^. This tool contains information on approximately 54,000 genes and survival data for 21 different types of cancers. We analyzed the impact of these genes on the overall survival (OS) of patients with breast cancer. A log-rank P-value of less than 0.05 was considered statistically significant.

### Validation of prognostic WNTs using GEO and correlation analysis

We queried the NCBI GEO database using “breast tumor” as a keyword, selecting original experimental studies that profiled both tumor and normal breast tissues. Our inclusion criteria for the datasets used in our study were as follows: (i) the samples in the datasets were from “Homo sapiens”; (ii) the datasets were " Expression profiling by high throughput sequencing”; (iii) the dataset submission date to GEO was within the last 12 years (i.e., 2012–2024); (iv) for each dataset, the total number of available samples was ≥ 30; (v) the samples from the studies included both tumor and healthy controls; and (vi) both raw and processed data of the datasets were available. Furthermore, we excluded abstracts, case reports, review articles, scRNA-seq studies, studies using cell line-based experimental designs, and studies lacking healthy controls or using nonhuman samples from our query. We then downloaded the data using the GEOquery (version 2.74.0) R package and performed differential gene expression analysis using the DESeq2 (v1.46.0) R package^27^. We used the EnhancedVolcano (version 1.24.0) R package to visualize the most significant differentially expressed genes (DEGs), with specified thresholds of log2(fold change) (log2FC) greater than |2| and an adjusted P-value cut-off of 10e-6^28^. The presence of key prognostic WNTs was verified based on the list of differentially expressed genes. Next, we utilized the TCGAplot (v8.0.0) R package to perform gene-gene correlation analysis of key prognostic WNTs across TCGA-BRCA tumors and corresponding normal tissues^29^. The complete workflow for this step, including all codes and documentation, is available at GitHub: https://github.com/bigbiolab/WNT_BRCA.

### Gene effect scores for key WNTs in BRCA cell lines

We acquired breast cancer cell lines along with gene effect score data processed by the Chronos algorithm from UALCAN, which is derived from genome-wide CRISPR knockout screens published by Broad’s Achilles and Sanger’s SCORE projects in DepMap^24,30^. We then visualized the processed data using the ggplot2 (version 3.5.1) R package^31^.

### DNA methylation analysis of WNTs in BRCA patients

The Shiny Methylation Analysis Resource Tool (SMART; http://www.bioinfo-zs.com/smartapp/) was used to generate a tumor vs. Normal Methylation Box Plot to visualize differential methylation patterns between the tumor and normal samples^32^. The “Methylation Box Plot” option was selected from the “Methylation DIY” module, and the BRCA dataset was chosen. The methylation value was configured as a beta value, and the aggregation method was set to the median. Wilcoxon tests confirmed statistically significant differences in methylation (*p* < 0.05) between BRCA tumors and normal tissues.

### Investigation of genetic alteration within BRCA cohorts

The genetic alteration status of WNT2, WNT7B, and WNT11 in patients with BRCA across various cancer cohorts was analyzed using cBioPortal for Cancer Genomics (https://www.cbioportal.org/)^33^. This investigation focused on breast cancer across 30 studies available on cBioPortal that evaluated alteration frequency, mutation types, mutations, structural variants, amplifications, deep deletions, multiple alterations, and copy number alterations (CNA). The WNT2, WNT7B, and WNT11 alteration frequencies and mutation types in TCGA tumors were examined using the “Cancer Types Summary” module of cBioPortal. The “Mutations” module was employed to obtain a detailed overview of gene mutations. We obtained the 3D structures of the proteins using AlphaFold^34^.

### Immune filtration assessment

We investigated the correlations between WNT2, WNT7B, and WNT11 expression, and immune cell ratio and immune score via Pearson’s method utilizing the TCGAplot (version 8.0.0) (https://github.com/tjhwangxiong/TCGAplot) R package and visualized them using heatmaps^29^. Additionally, we sorted out the significant and common positively correlated ( *p* < 0.05) chemokines, chemokine receptors, immune checkpoint genes (ICGs), immune inhibitors, and immune stimulators from the heatmaps generated by the TCGAplot R package and visualized them using the ggVennDiagram (v1.5.3) R package^35^.

### Single-cell analysis

We used the “Gene” module of Tumor Immune Single-cell Hub 2 (TISCH2) (http://tisch.comp-genomics.org/), a scRNA-seq database with detailed cell-type annotation at the single-cell level across different cancer types, to investigate the expression of WNTs in different cell-types within the tumor microenvironment (TME) of BRCA^36^. We set the following configurations: (i) Gene: “WNT2, WNT7B, and WNT11”; (ii) Cell-type annotation: “Celltype(major-lineage)”; (ii) Cancer type: “BRCA (Breast Invasive Carcinoma)”; (iv) Lineage for calculating correlation: “All lineage”; (v) selected datasets associated with “Homo sapiens” to be used. We downloaded the log (TPM/10 + 1) expression of genes in different cell types across datasets as “CSV” and visualized the data using the ggplot2 (version 3.5.3) R package. We used the ggVennDiagram (v1.5.4) R package to visualize common cell types across WNTs in a Venn diagram.

### Drug sensitivity analysis

We employed the “Drug” module of Gene Set Cancer Analysis (GSCA)( https://guolab.wchscu.cn/GSCA) to perform drug sensitivity analysis^37^. We inputted genes acquired from the STRING database to obtain the correlation between drug sensitivity and mRNA expression using drug sensitivity information from the Genomics of Drug Sensitivity in Cancer (GDSC) and the Cancer Therapeutics Response Portal (CTRP). Furthermore, we used the ggplot2 ((v3.5.1) and ggpubr (v0.6.0) R packages to visualize the correlation between WNT2, WNT7B, and WNT11 expression and drug sensitivity using GDSC and CTRP data exported from GSCA after applying an false discovery rate (FDR) cut-off of < 0.1 for significance^38^.

### PPIN construction and enrichment analysis

The STRING database (https://​string-​db.​org/) was utilized to acquire the protein-protein interaction (PPI) networks of WNT2, WNT7B, and WNT11^39^. We input WNT2, WNT7B, and WNT11 in the Multiple proteins and set the following configurations in the “Basic Settings” of the “Settings” module: (i) Network type: full STRING network; (ii) meaning of network edges: evidence; (iii) active interaction sources: Text mining, Experiments, Databases, CO-expression, Neighborhood, Gene Fusion, Co-occurrence; (iv) minimum required interaction score: low confidence (0.150), max number of interactors to show (1st shell): no more than 50 interactors. The results were exported and visualized using the Cytoscape (v 3.10.3) software^40^. Thereafter, we used the retrieved interacting genes from STRING to perform enrichment analysis, including Gene Ontology (GO) enrichment analysis of molecular functions (MFs), cellular components (CCs), biological processes (BPs), and Kyoto Encyclopedia of Genes and Genomes (KEGG) using the clusterProfiler (v4.14.4) R package, and Reactome enrichment analysis through ReactomePA (v1.50.0)^41–43^. Pathways with an adjusted P-value < 0.05 (Benjamini-Hochberg (BH) corrected) were considered to be significantly enriched. The top 15 most significant pathways from each analysis were visualized using the enrichplot (v1.26.5) R package^44^.

### Statistical analysis

In UCSC XENA, a gene expression heatmap was generated using log2-transformed expression values, and a t-test was performed to compare the expression levels between different groups. Wilcoxon test was used to evaluate the differential expression of genes between tumors and adjacent normal tissues. The Log-rank test and Cox regression were used to calculate the HR and log-rank P-value in the Kaplan–Meier Plotter to analyze the overall survival curves. Pearson’s correlation was used to estimate correlations between gene expression and chemokines, chemokine receptors, immune checkpoint genes (ICGs), immune inhibitors, immune stimulators, immune cell ratio, and immune score. The Benjamini-Hochberg (BH) procedure was applied to account for the adjusted P-value in the pathway enrichment analysis and the false discovery rate (FDR) in the drug sensitivity analysis. Results with p-adjust < 0.05 and FDR < 0.10 were considered statistically significant. All statistical analyses were conducted using R (v4.4.2) with RStudio software (v2024.12.0 + 467)^45^. A P-value of less than 0.05 was set as the significance threshold for all statistical analyses.

## Results

### Analysis of the expression patterns of WNTs in breast cancer

**Fig. 1:**
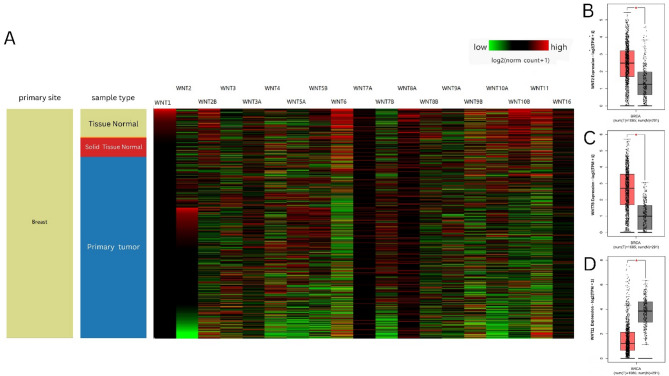
Expression pattern of WNTs in Breast cancer. **A** mRNA expression pattern of WNTs in breast cancer patients. Heat Map displaying the expression patterns of WNTs using UCSC XENA. Box-and-whisker plots displaying the relative mRNA expression levels of **B** WNT2, **C** WNT7B, **D** WNT11, across TCGA-BRCA and normal samples. Grey-and red-colored box areas signify normal and tumor patient samples. *P < 0.05.)

First, we used the UCSC XENA database to examine the expression patterns of WNTs in BRCA patients. Our study revealed substantial WNT deregulation in BC (Fig. 1A). In addition, we used the GEPIA2 database, which displayed the log2 fold change of different WNTs in BRCA. WNT2 and WNT7B were upregulated, with log2 fold changes of 1.237 and 1.712, respectively. In contrast, WNT11 was downregulated, with a log2 fold change of − 2.639 and a P-value of 4.07E-74 (Table 2). The relative mRNA expression distribution across the TCGA-BRCA cohort was compiled using GEPIA2 and displayed as WNT2 (Fig. 1B). WNT7B (Fig. 1C) expression levels were significantly upregulated. Conversely, WNT11 expression was significantly downregulated in tumor samples compared with that in the corresponding control tissue, as shown by box-and-whisker plots (Fig. 1D).

**Table 2:** Log2 fold change of WNTs in breast cancer.

Gene symbol	Gene ID	Median (tumor)	Median (normal)	Log2 (fold change)	adjp
WNT1	ENSG00000125084.11	0.000	0.020	-0.029	5.19e-13
**WNT2**	**ENSG00000105989.8**	**4.610**	**1.380**	**1.237**	**5.65e-25**
WNT2B	ENSG00000134245.17	0.520	2.100	-1.028	4.96e-87
WNT3	ENSG00000108379.9	1.970	2.110	-0.066	4.15e-1
WNT3A	ENSG00000154342.5	0.020	0.030	-0.014	3.64e-1
WNT4	ENSG00000162552.14	2.720	1.370	0.650	3.42e-7
WNT5A	ENSG00000114251.13	4.080	2.820	0.411	2.02e-3
WNT5B	ENSG00000111186.12	2.930	4.020	-0.353	1.16e-12
WNT6	ENSG00000115596.3	0.190	1.920	-1.295	1.80e-34
**WNT7B**	**ENSG00000188064.9**	**5.520**	**0.990**	**1.712**	**2.35e-50**
WNT8B	ENSG00000075290.7	0.040	0.060	-0.027	3.57e-3
WNT9A	ENSG00000143816.7	2.930	2.170	0.310	1.19e-7
WNT9B	ENSG00000158955.10	0.060	0.180	-0.155	3.37e-24
WNT10B	ENSG00000169884.13	0.210	0.560	-0.367	2.12e-28
**WNT11**	**ENSG00000085741.12**	**1.320**	**13.450**	**-2.639**	**4.07e-74**
WNT16	ENSG00000002745.12	0.030	0.040	-0.014	6.80e-1

### WNTs expression based on clinicopathological characteristics of breast cancer

**Fig. 2:**
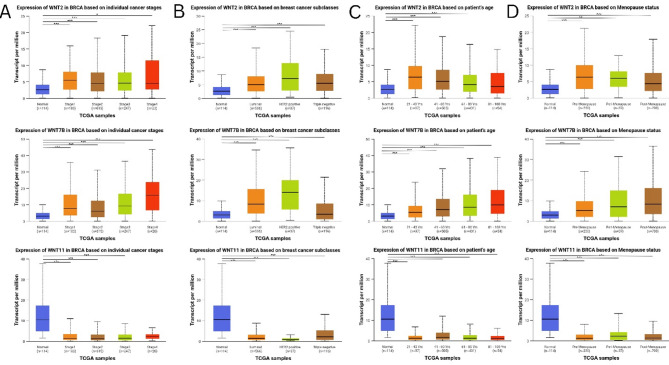
Expression pattern of highly deregulated WNTs in BC based on clinicopathological characteristics by UALCAN database. Expression pattern of WNT2, WNT7B, WNT11 based on **A** cancer stages, **B** major subclasses, **C** patient age, **D** menopause status. *P < 0.05; **P < 0.01; ***P < 0.001.

UALCAN database analysis revealed the expression pattern of highly deregulated WNTs among cancer stages, major subclasses, patient age, and menopausal status in BRCA. Both WNT2 and WNT7B showed elevated expression levels across individual cancer stages; in particular, WNT2 had higher expression at stage 1, whereas WNT7B had higher expression at stage 4 compared to normal tissues. Conversely, lower expression of WNT11 was observed across individual cancer stages; specifically, stage 2 displayed the lowest expression compared with that in normal tissue (Fig. 2A). Similarly, WNT2 and WNT7B were highly upregulated, whereas WNT11 was highly downregulated in HER2-positive breast tumors compared with that in the control tissue (Fig. 2B). WNT2 was highly upregulated in women age 21–40 years, followed by WNT7B, which showed elevated expression at 81–100 years, whereas WNT11 showed lower expression at 81–100 years (Fig. 2C). In addition, WNT2 and WNT7B were highly upregulated in the premenopausal and postmenopausal stages; in contrast, WNT11 was highly downregulated in the premenopausal stage (Fig. 2D).

### Pan-cancer view of the WNT family

**Fig. 3:**
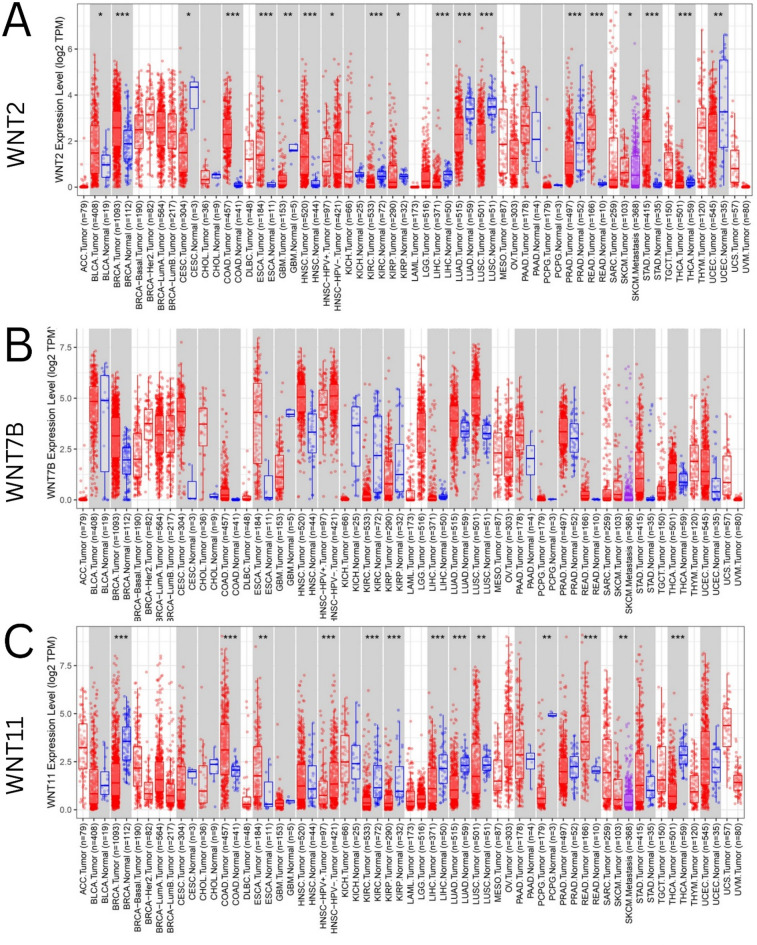
Pan-cancer analysis of highly deregulated WNTs utilizing TIMER2.0. **A** Expression levels of WNT2 in different tumors vs. corresponding controls. **B** Expression levels of WNT7B in different tumors vs. corresponding controls. **C** Expression levels of WNT11 in different tumors vs. corresponding controls. (*P < 0.05; **P < 0.01; ***P < 0.001)

To investigate the patterns of WNT2, WNT7B, and WNT11 expression in various cancer types, we used TIMER2.0, to explore the expression levels of WNT2, WNT7B, and WNT11 in 33 cancer types and matched normal pairs from the TCGA and GTEx databases. The expression level of WNT2 was significantly lower in tumors than in the corresponding normal tissues, including CESC, HNSC-HPV positive, KIRP *(P* < 0.05), GBM, UCEC *(P* < 0.01), KIRC, LIHC, LUAD, LUSC, PRAD, and THCA (*P* < 0.001), than in the corresponding control tissues. In contrast, the expression level of WNT2 was significantly higher in BLCA, SKSM (*P* < 0.05), BRCA, COAD, ESCA, NHSC, READ, and STAD (*P* < 0.001) than in matched adjacent normal tissues (Fig. 3A). Furthermore, the expression levels of WNT7B were significantly higher than those in the matched adjacent healthy tissues, including CESC *(P* < 0.01), BRCA, CHOL, COAD, ESCA, GBM, LUAD, LUSC, READ, SKSM, STAD, THCA, and UCEC (*P* < 0.001). Interestingly, the expression level of WNT7B was significantly lower in HNSC-HPV-positive, KIHC (*P* < 0.01), KIRC, LIHC, and NHSC (*P* < 0.001) tissues than that in the corresponding healthy tissues (Fig. 3B). The expression level of WNT11 was significantly lower in tumors than in corresponding normal tissues, including BRCA, HNSC-HPV-positive, KIRC, KIRP, LIHC, LUAD (*P* < 0.001), LUSC, and PCPG (*P* < 0.01). In contrast, the expression level of WNT11 was significantly higher in SKSM, ESCA (*P* < 0.01), COAD, READ, and THCA (*P* < 0.001) than in matched normal tissues (Fig. 3C).

### Survival prognosis analysis across the BRCA cohort

**Fig. 4:**
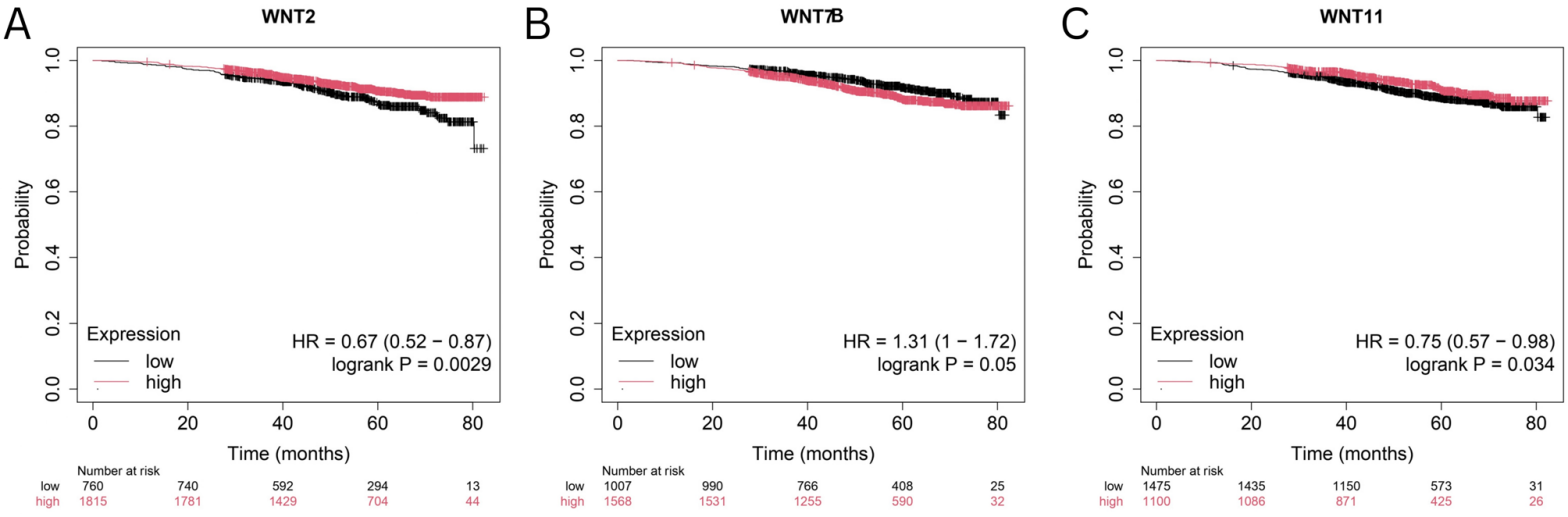
Expression of WNTs correlates with survival outcomes in BC patient. KM plots showing the OS of **A** WNT2, **B** WNT7B, **C** WNT11. Red and black colors signify higher and lower expression groups.

The Kaplan–Meier Plotter tool was used to explore the correlation between WNTs expression levels and the prognosis of BRCA patients. According to the overall survival module, the analysis revealed that higher expression of WNT2 was significantly associated with a better prognosis than lower expression (OS: HR = 0.67, *P* = 0.0029) (Fig. 4A), whereas WNT7B displayed a substantially poorer prognosis with elevated expression, indicating its oncogenic potential in BRCA progression (OS: HR = 1.34, *P* = 0.035) (Fig. 4B). Interestingly, following WNT2, elevated expression of WNT11 was associated with a better survival rate than lower expression (OS: HR = 0.75, *P* = 0.033) (Fig. 4C). These findings highlight that WNT2 and WNT11 may serve as protective and favorable prognostic biomarkers, respectively. Although WNT7B may have detrimental effects, it could be a potential target for therapeutic intervention in BRCA.

### Validation of prognostic WNTs using GEO and correlation analysis

**Fig. 5:**
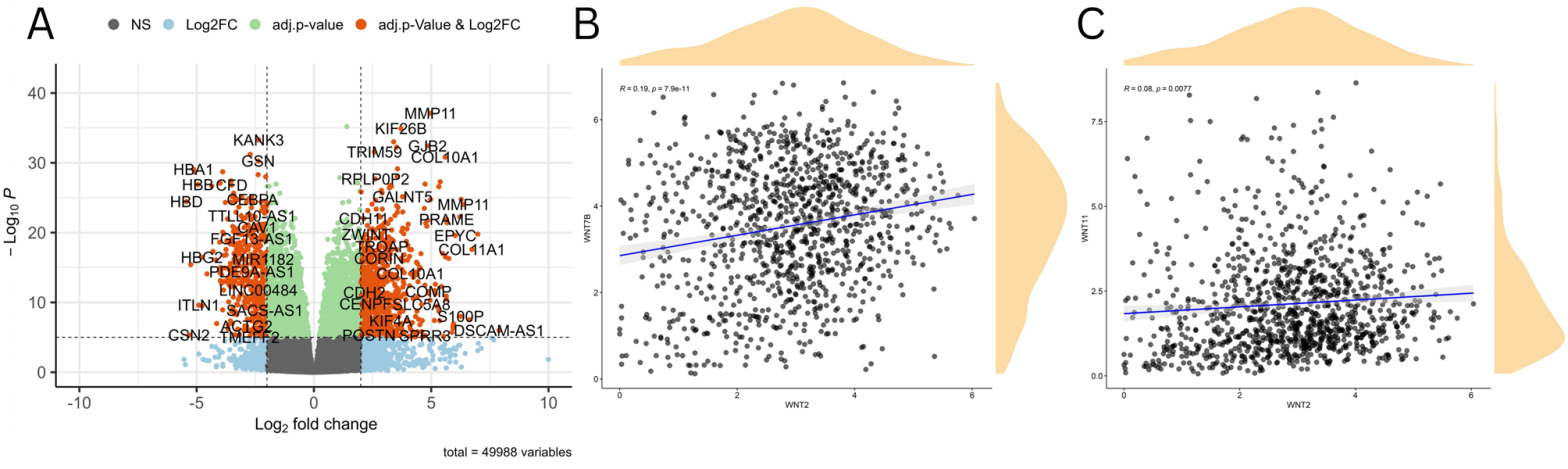
**A** Visualization of the most significant DEGs in GEO datasets. Scatterplots showing Spearman pairwise correlations between **B** WNT2 and WNT7B, **C** WNT2 and WNT11.

Based on the defined inclusion and exclusion criteria, we selected BRCA-associated expression profiling using high-throughput sequencing datasets GSE233242 (42 healthy controls and 42 tumor tissues) and GSE227679 (16 healthy controls and 16 tumor tissues) for further analysis. The analysis yielded 12,792 differentially expressed genes (DEGs) from GSE233242 and 1882 DEGs from GSE227679 (Supplementary Table S1). Importantly, all critical prognostic WNT genes, namely, WNT2, WNT7B, and WNT11, were consistently identified within the DEG lists from both datasets, corroborating their validation in the external GEO datasets. WNT2 and WNT7B were upregulated, whereas WNT11 was downregulated in the DEG list, which aligned with the preliminary results derived from GEPIA 2. A volcano plot was used to visualize the most significant DEGs (Fig. 5A). Scatter plots display pairwise correlations among key prognostic WNT genes. A significant positive correlation was observed between WNT2 and WNT7B (*R* = 0.19, P-value = 7.9 × 10 − 11) (Fig. 5B) and between WNT2 and WNT11 (*R* = 0.08, P-value = 0.0077) (Fig. 5C).

### Gene effect scores for key WNTs in BRCA cell lines

**Fig. 6:**
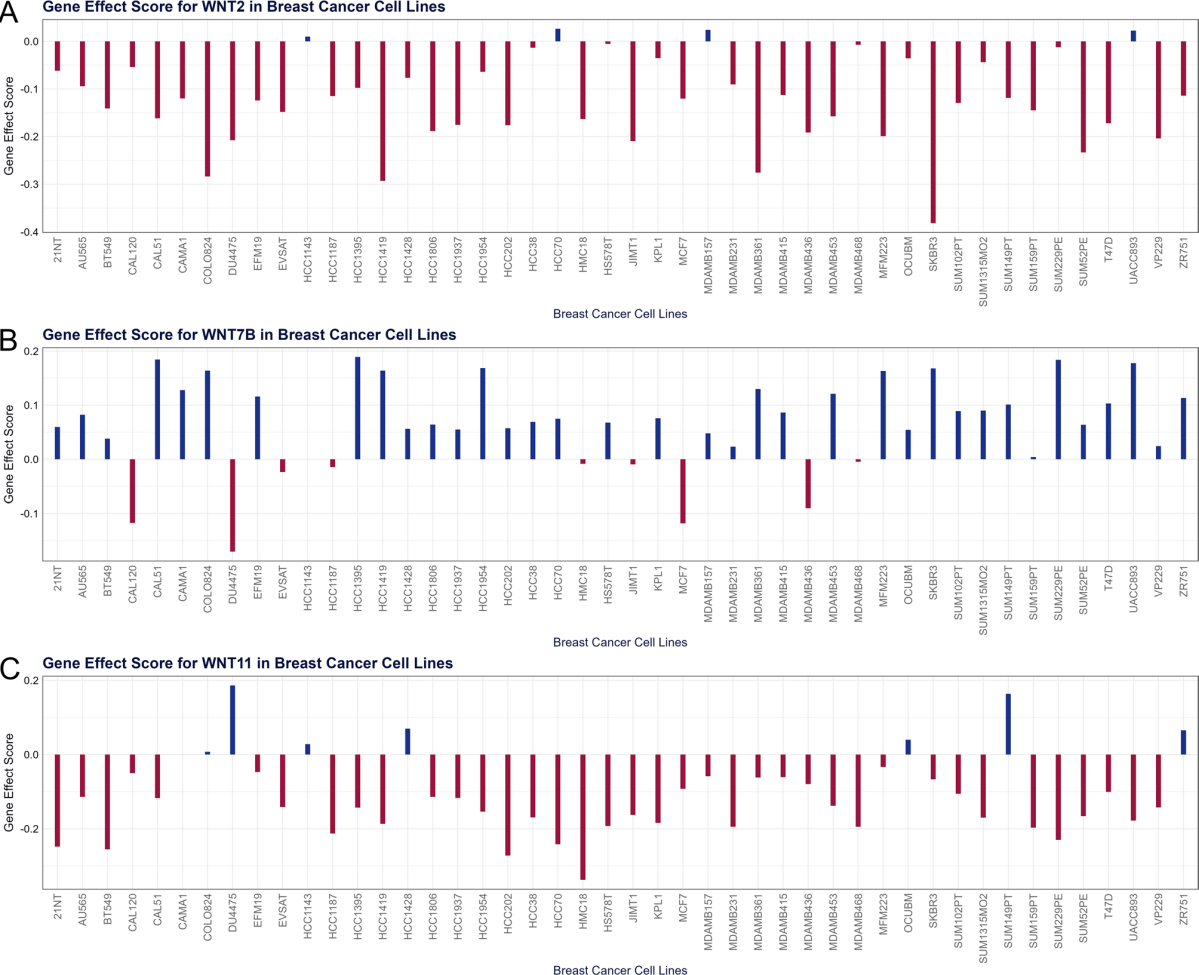
Gene effect scores for **A** WNT2, **B** WNT7B, and **C** WNT11 in various breast cancer cell lines.

Gene effect scores were assessed across several breast cancer cell lines to determine whether key WNTs were crucial in BRCA progression. A negative gene effect score indicated that the cell line was highly dependent on the gene for survival as gene depletion reduced cell viability. Conversely, a positive score reflects minimal dependency on the gene, with a minor impact on survival upon depletion. Bar plot analysis revealed that WNT2 and WNT11 exhibited negative gene effect scores in most cell lines, whereas WNT7B exhibited negative gene effect scores in a few cell lines (Fig. 6). Additionally, all these genes showed a negative gene effect score in MCF7, HCC1187, CAL120, MDAMB436, JIMT1, HMC18, MDAMB468, and EVSAT breast cancer cell lines, as visualized using a Venn diagram (Supplementary Fig. 1).

### DNA methylation analysis of WNTs in BRCA patients

**Fig. 7:**
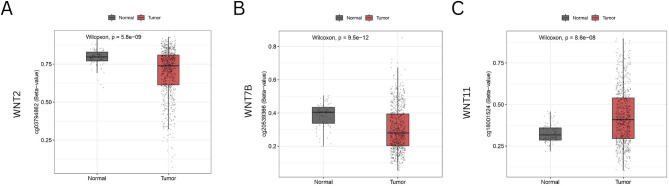
The methylation levels of **A** WNT2, **B** WNT7B, and **C** WNT11 between normal tissues and tumor tissues using the SMART database.

DNA methylation, a pivotal epigenetic mechanism, plays a crucial role in the onset and progression of various forms of cancer. We investigated 20 probes within WNT2, 28 probes within WNT7B, and 31 probes within WNT11, to evaluate the methylation levels of these specific genes. Compared to normal tissues, WNT2 and WNT7B exhibited lower methylation levels (Fig. 7A, B), whereas WNT11 showed higher methylation in BRCA tumors (Fig. 7C). The probes cg03794862, cg20539366, and cg18001524 revealed significant levels of methylation within the WNT2, WNT7B, and WNT11 genes, respectively.

### Investigation of genetic alteration within BRCA cohorts

**Fig. 8:**
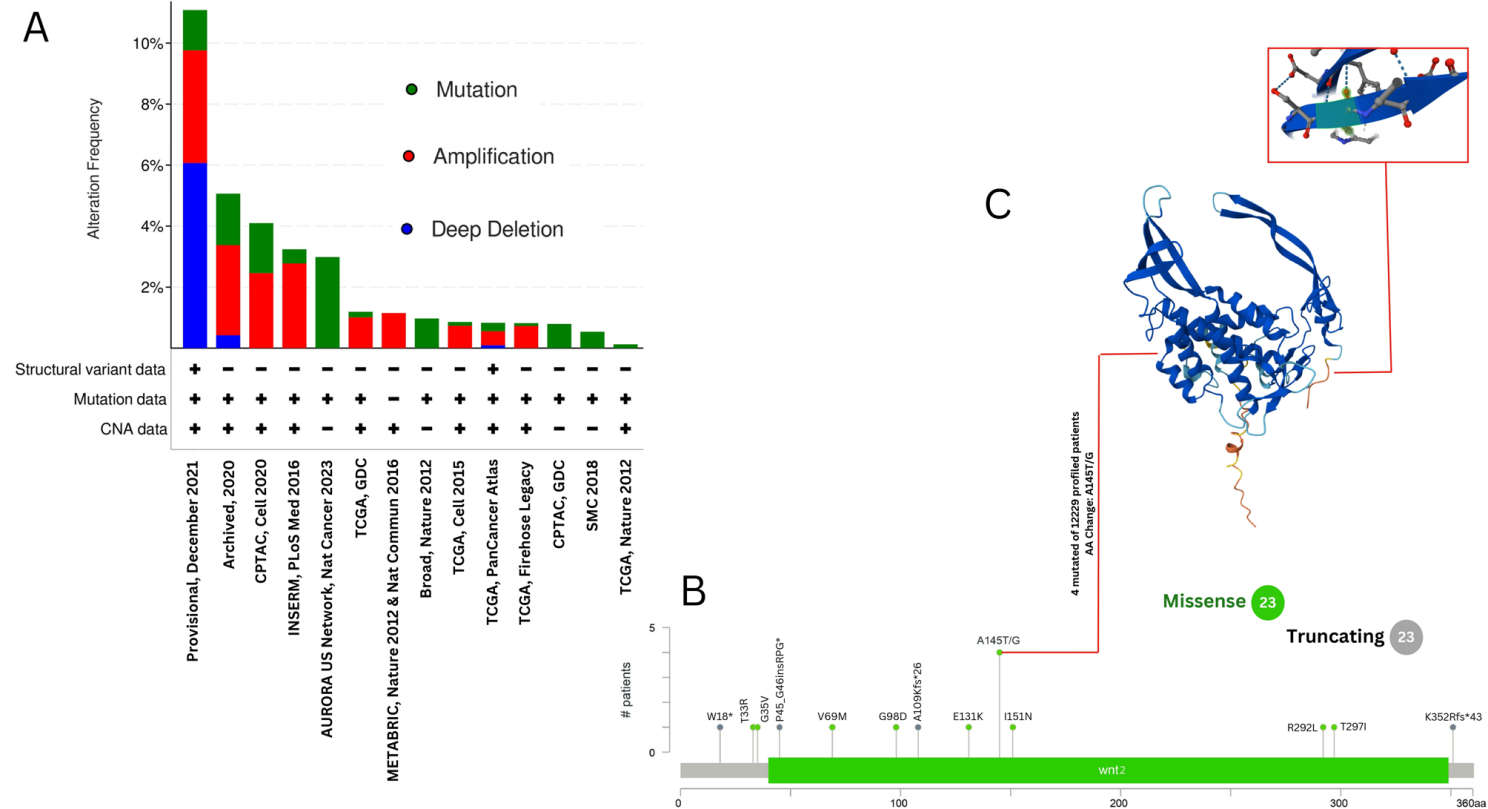
The genetic alterations of WNT11 across the BRCA cohort were analysed by the cBioPortal database. **A** Alterations summary of WNT11 **B** The mutation types, number, and sites of the WNT11 genetic alterations. **C** 3D protein structure of WNT11 from AlphaFold.

Investigation of the genetic alteration status of WNT2, WNT7B, and WNT11 in patients with BRCA across various cancer cohorts using cBioPortal has revealed notable insights. Among the 12,148 patients analyzed, WNT2 alterations were observed in 128 individuals (1%), showing diverse alterations with frequencies ranging from 0.12 to 11.08% (Supplementary Fig. 2A). Similarly, WNT7B mutations were identified in 173 patients (1%) of the total cohort. Distinct copy number alterations were identified, ranging from 0.37 to 28.23% in frequency (Supplementary Fig. 3A). As observed, WNT11 displayed a maximum frequency of genetic alterations in 609 patients (5%), showing diverse alterations with frequencies ranging from 0.54 to 37.73% (Fig. 8A). Our analysis highlighted “amplification” as a common genetic alteration across various BRCA cohorts. The highest frequency rate of “amplification” of WNT2, WNT7B, and WNT11 was recorded at 3.69%, 15.83%, and 33.25%, respectively, notably within The Metastatic Breast Cancer Project (Provisional, December 2021). Missense mutations were the main type of WNT genetic mutation, and the most frequent mutations were A145T/G (Supplementary Fig. 2B), A176T (Supplementary Fig. 3B), and V69A (Fig. 8B) in WNT2, WNT7B, and WNT11, respectively. The 3D structures of key WNTs proteins were predicted using AlphaFold, as shown in (Supplementary Fig. 1C, Supplementary Figs. 2 C, 8 C).

### Correlation between WNTs and immune microenvironment in breast cancer

We explored whether gene expression was related to the level of immune infiltration in BRCA. Immune and stromal cells play essential roles in regulating the development and progression of cancers, accounting for significant components of the tumor microenvironment (TME), and their infiltration levels influence immunotherapy efficacy. WNT2 and WNT11 were significantly positively linked to the immune scores in patients with BRCA (Supplementary Fig. 4). WNT2 expression was negatively correlated with the maximum number of immune cells, including resting mast cells, monocytes, resting NK cells, plasma cells, activated dendritic cells, memory B cells, naïve CD4 T cells, activated NK cells, T cells CD8, follicular helper T cells, and eosinophils (Supplementary Fig. 5). Similarly, WNT7B negatively correlated with memory-activated CD4 T cells, naïve B cells, activated NK cells, follicular helper T cells, plasma cells, memory B cells, and naïve CD4 T cells (Supplementary Fig. 6). In contrast, WNT11 expression positively correlated with follicular helper T cells, activated dendritic cells, and naïve B cells (Supplementary Fig. 7).

**Fig. 9:**
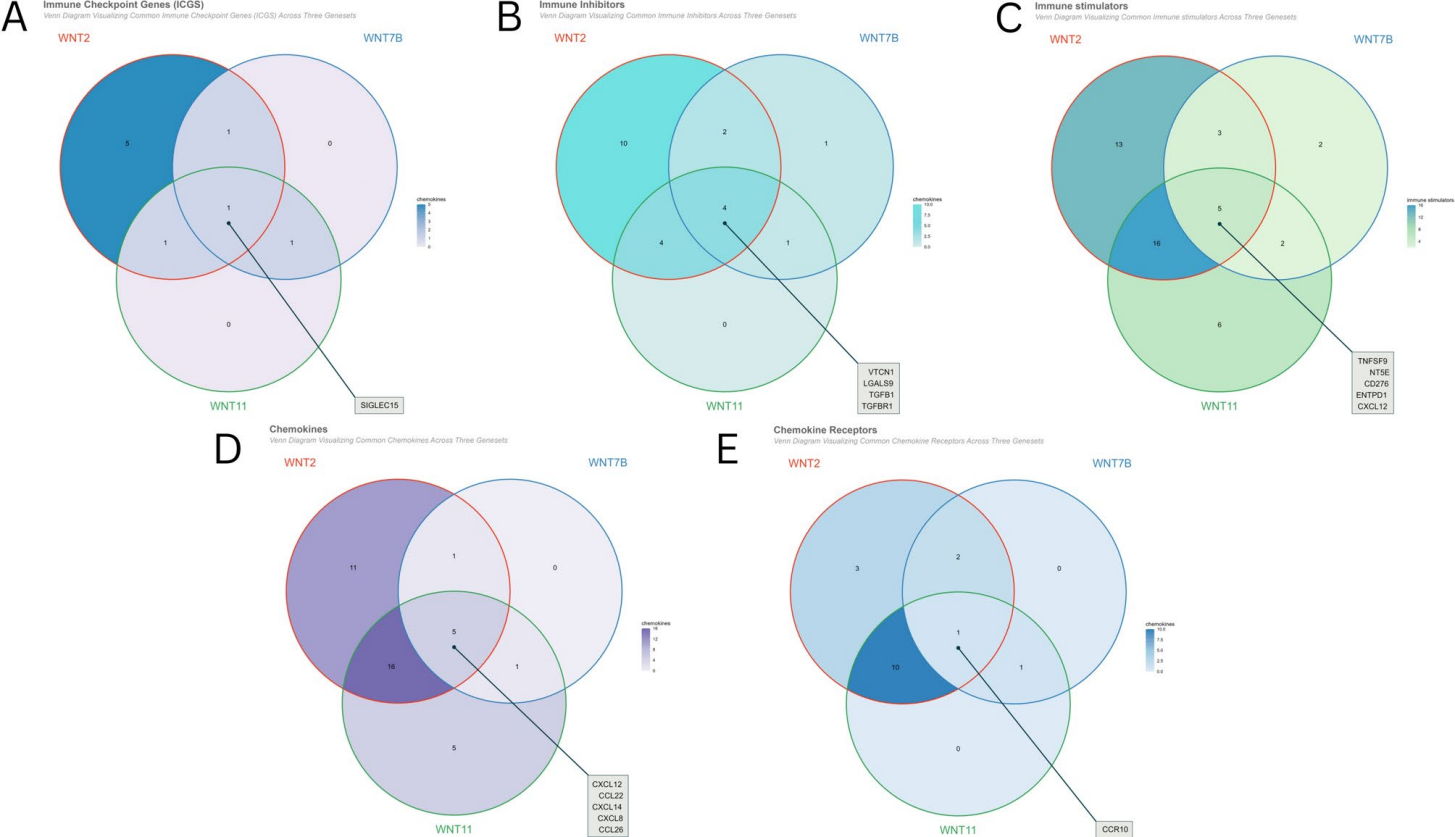
Visualization of typical patterns of **A** immune checkpoint genes, **B** immune inhibitors, **C** immune stimulators, **D** chemokines, **E** chemokine receptors among WNT2, WNT7B, and WNT11.

The correlation between the expression of WNTs and immune-related genes was visualized using heat maps (Supplementary Fig. 8–12). All these genes were positively correlated with the immune checkpoint gene SIGLEC15 (Fig. 9A), immune inhibitory genes VTCN1, LGALS9, TGFB1, and TGFBR1 (Fig. 9B), and immunostimulatory genes TNFSF9, NT5E, CD276, ENTPD1, and CXCL12 (Fig. 9C). All of these genes were positively correlated with the chemokines CXCL12, CCL22, CXCL14, CXCL8, and CCL26 (Fig. 9D) and the chemokine receptor CCR10 (Fig. 9E). This comprehensive correlation underscores the wide-ranging influence of WNT’s expression on immunity in BRCA.

### Single-cell analysis

**Fig. 10:**
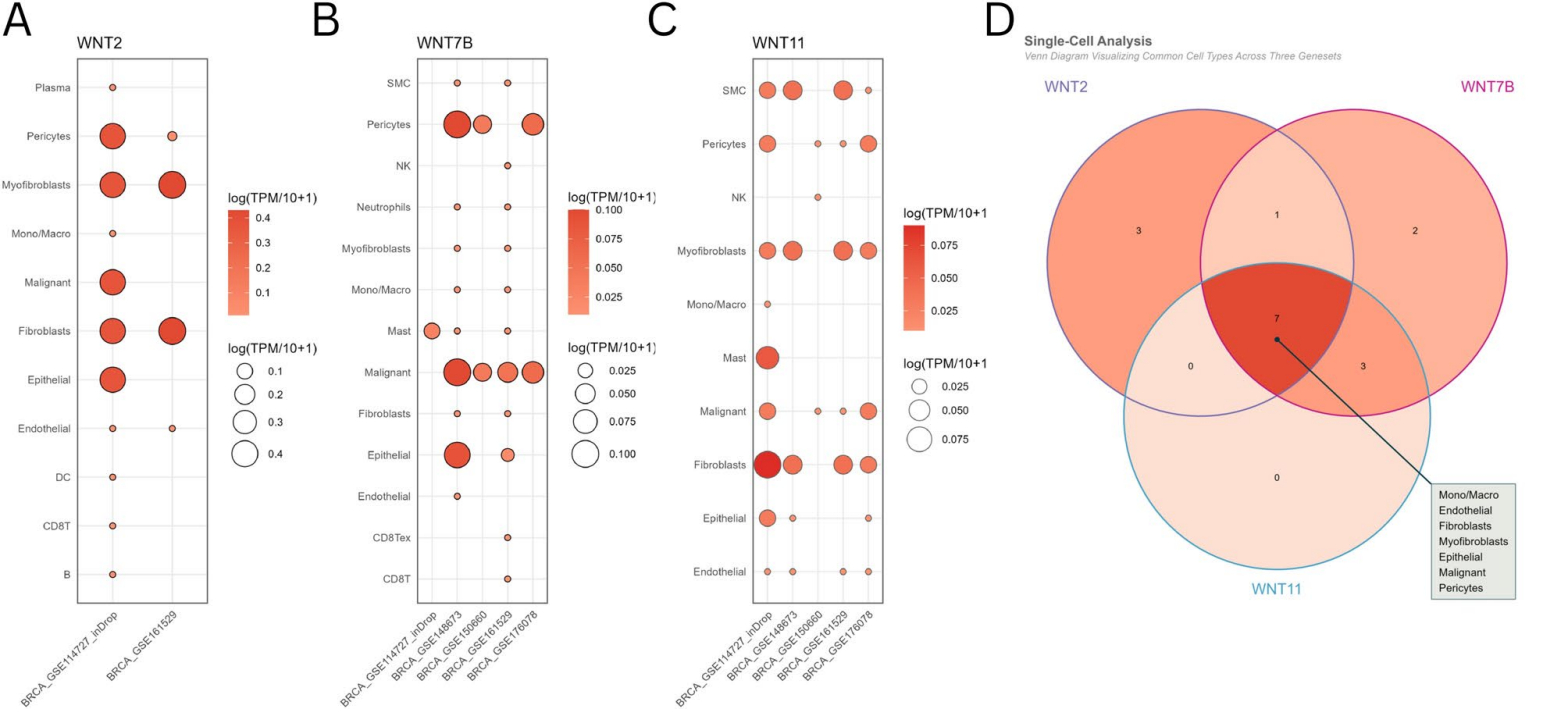
Expression analysis of key WNTs from scRNA sequencing database. **A** expression of WNT2 in subgroups of immune cells. **B** expression of WNT7B in subgroups of immune cells. **C** expression of WNT11 in subgroups of immune cells. **D** Visualization of typical patterns of immune cells among WNT2, WNT7B, and WNT11.

The tumor microenvironment comprises a heterogeneous collection of immune, stromal, and cancerous cells. We used TISCH2, an scRNA-seq database focusing on the TME, to provide detailed cell-type annotation and gene expression at the single-cell level in BRCA. We found that WNT2 was highly expressed in myofibroblasts and fibroblasts (Fig. 10A). WNT7B was highly expressed in the malignant cells and pericytes (Fig. 10B). WNT11 was highly expressed in the fibroblasts (Fig. 10C). Setting the value of log (TPM/10 + 1) > 0, we found that all of these genes were expressed in Mono/Macro, epithelial, malignant, myofibroblasts, endothelial cells, fibroblasts, and pericytes, as visualized by the Venn diagram (Fig. 10D).

### Drug sensitivity analysis of WNTs

**Fig. 11:**
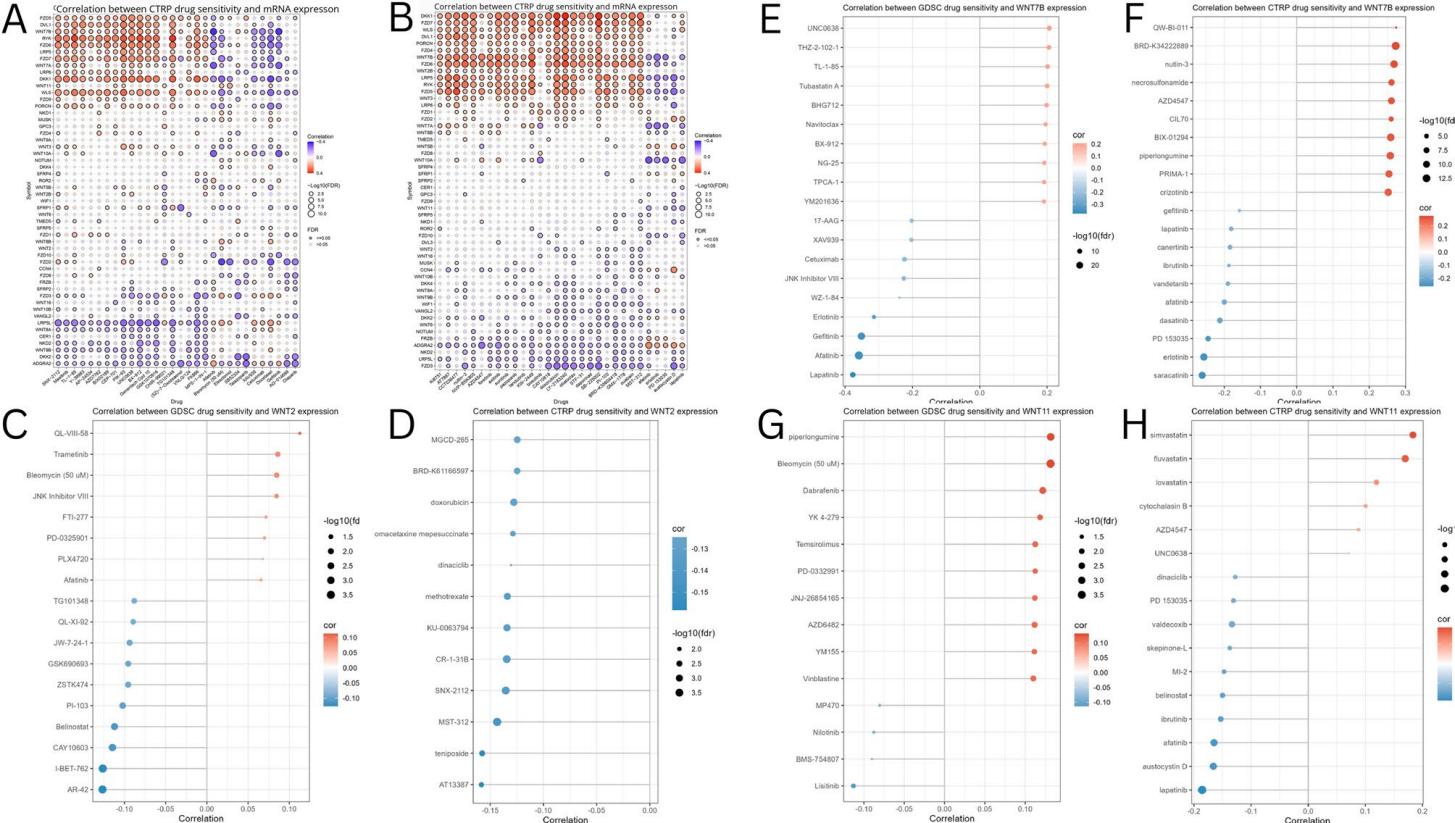
Key WNTs expression predicts drug sensitivity. The correlation between WNTs and 53 identified interacting genes and their drug sensitivity **A** by GDSC, **B** by CTRP. The correlation between WNT2 expression and the most significant drug sensitivity **C** by GDSC, **D** by CTRP. The correlation between WNT7B expression and the most significant drug sensitivity **E** by GDSC, **F** by CTRP. The correlation between WNT11 expression and the most significant drug sensitivity **G** by GDSC, **H** by CTRP.

These findings suggest the involvement of WNT genes in BRCA prognosis and immune responses. We further investigated the potential links between WNTs and the 53 identified interacting genes and their sensitivity to drugs using GDSC and CTRP databases. Our analysis revealed the strongest positive correlation between WNT7B, and genes co-expressed with 19 and 24 anti-cancer drugs, and a negative correlation with 6 and 4 drugs, respectively (Fig. 11A, B).

Correlations between the individual expression of WNT2, WNT7B, and WNT11 and drug sensitivity were visualized using the ggpubr package. Notably, the results from the GDSC database indicated that WNT2 expression was most positively correlated with QL-VIII-58 and negatively correlated with AR-42 (Fig. 11C). WNT7B demonstrated the strongest positive correlation with UNC0638, in contrast to the negative correlation with lapatinib (Fig. 11E). WNT11 showed the strongest positive correlation with Piperlongumine, while exhibiting a negative correlation with lestaurtinib (Fig. 11G). Furthermore, findings from the CTRP database revealed that WNT2 was negatively correlated with all the anti-cancer drugs (Fig. 11D). WNT7B exhibited the strongest positive correlation with QW-BI-011 but was negatively correlated with saracatinib (Fig. 11F). WNT11, on the other hand, was associated with the most positive correlations with simvastatin and the most negative correlations with lapatinib (Fig. 11H). These results suggest that WNT genes may serve as valuable biomarkers for cross-cancer drug screening, thereby facilitating the identification of effective therapeutic strategies.

### PPIN construction and enrichment analysis

**Fig. 12:**
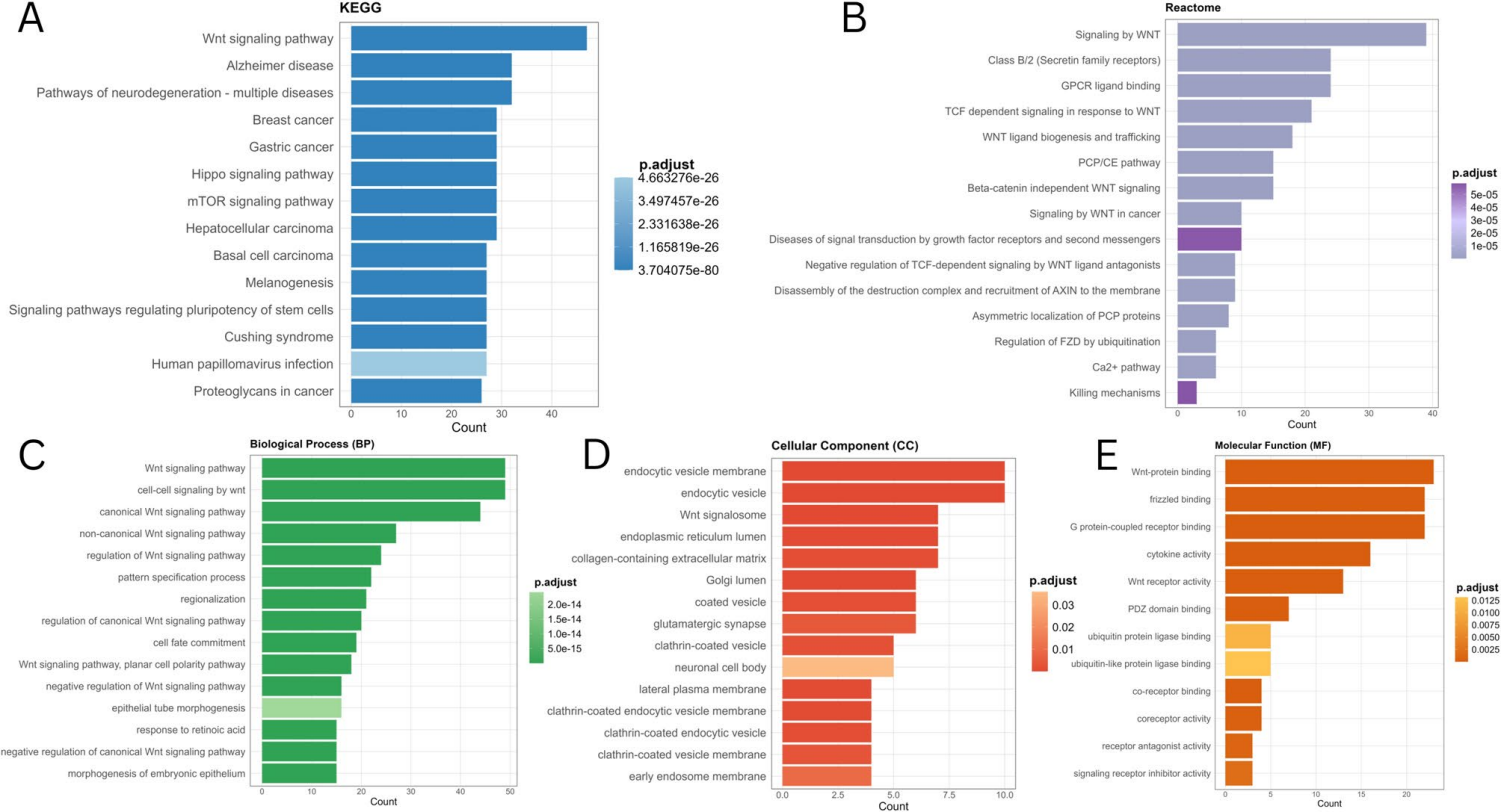
Enrichment analysis of the related genes of WNT2, WNT7B, and WNT11. **A** KEGG pathway, **B** Reactome pathway, **C** GO-BP, **D** GO-CC, and **E** GO-MF analysis.

We screened the proteins interacting with WNT2, WNT7B, and WNT11 using the STRING online tool to explore the molecular mechanisms of tumorigenesis. We found 53 proteins that were supported by experimental evidence, and the interaction network of these genes is shown (Supplementary Fig. 13). Our PPIN consists of 53 nodes and 1234 edges. Within the PPIN, node degrees ranged from 16 to 56, betweenness ranged from 1 to 63.966, and closeness ranged from 0 to 1. The average degree, betweenness, and closeness values for the PPIN were 46.576, 33.802, and 0.947, respectively. Topological/centrality measures for the PPIN, including node degree, betweenness, closeness, clustering coefficient, neighborhood connectivity, and average shortest path length, are presented in Supplementary Tables S2 and S3. Subsequently, we utilized the gene set to perform Kyoto Encyclopedia of Genes and Genomes (KEGG), Reactome pathway, and Gene Ontology (GO) analyses of key prognostic WNTs. KEGG pathway analysis correlated with critical pathways, notably “Alzheimer’s disease,” “Pathways of neurodegeneration,” “Breast & Gastric cancer,” “Hippo signaling,” and “mTOR signaling pathway” mTOR signaling pathways (Fig. 12A). Reactome pathway enrichment analysis showed that they are primarily involved in “class B/2,” “GPCR ligand binding,” “TCF-dependent signaling,” and “PCP/CE pathway” (Fig. 12B). Both pathways were highly enriched in the “Wnt signaling pathway.” In addition, GO analysis showed that the genes were highly enriched in the “cell-cell signaling” (Fig. 12C), “endocytic vesicle membrane” (Fig. 12D), and “frizzled binding” (Fig. 12E) pathways in the BP, CC, and MF.

## Discussion

This study highlights the complex roles of the WNT gene family and potential treatment strategies for BRCA. The WNT family encodes signaling proteins crucial for regulating various cellular functions, including survival, proliferation, migration, and stem cell renewal^46^. Abnormal activation of the WNT pathway has been identified as a predisposing factor in various cancers and plays a significant role in CSC biology^47^. Specifically, WNT2 activates the canonical WNT/β-catenin pathway involved in colorectal and hepatocellular carcinoma^48^WNT7B promotes angiogenesis and is linked to several squamous cell carcinomas, and WNT11 operates through non-canonical pathways (WNT/PCP and WNT/Ca²⁺), contributing to cell polarity and metastasis, particularly in prostate cancer^12,16,49^. Despite the discovery of WNTs in mammary cancer nearly four decades ago, their comprehensive role in breast cancer development and progression has yet to be thoroughly investigated^16,49^. Our study bridges this gap by utilizing comprehensive multi-omics to reveal the molecular mechanisms driving tumorigenesis, offering new and promising avenues for targeted therapies, and revolutionizing treatment strategies for patients with BRCA.

This study identified consistent expression patterns of the WNT family in BRCA, with WNT2 and WNT7B being significantly upregulated and WNT11 being downregulated, corroborating previous research findings. Validation using two independent BRCA-associated GEO datasets confirmed the differential expression of these three genes, reinforcing their robustness as potential biomarkers for breast cancer^15,50^. Clinicopathological analysis revealed marked upregulation of WNT2 and WNT7B in HER2-positive breast cancer, suggesting a potential synergy between WNT signaling and HER2-driven oncogenic pathways. This association may offer a novel therapeutic avenue in which dual targeting of the WNT and HER2 pathways could enhance treatment efficacy in HER2⁺ patients. However, further preclinical and clinical validation are necessary to assess the feasibility of this combinatorial approach. Additionally, pan-cancer analysis highlighted the tissue- and tumor-specific expression patterns of WNT2, WNT7B, and WNT11 across various cancers, in agreement with previous studies^7,10,12,46,50,51^. These findings underscore the need for future functional studies to unravel the context-dependent roles of these WNT genes in different tumor environments.

Our results showed that WNT2’s elevated expression was associated with a significantly better prognosis, which contradicts its typical oncogenic role. However, previous studies have demonstrated that specific oncogenes can paradoxically activate protective mechanisms by promoting (i) Oncogene-induced Senescence (OIS)^52^ which limits unchecked proliferation and induces stable growth arrest; (ii) feedback loops leading to Tumor Suppression^53^where oncogene activation triggers compensatory anti-tumor pathways; (iii) non-oncogenic addiction^54^where cancer cells become dependent on specific pathways, making them more susceptible to targeted therapies; and (iv) Immunogenic Modulation of TME^55^potentially enhancing immune recognition and anti-tumor responses. Further investigations are required to understand these mechanisms, which may provide a predictive basis for developing novel drug combinations. Consistent with its proposed tumor-suppressive role, WNT11 overexpression has also been linked to improved survival outcomes. Conversely, WNT7B overexpression was linked to worse prognosis, which is consistent with its suggested oncogenic role^56^.

DNA methylation is a crucial epigenetic modification that enhances the stability of the transcriptional repression associated with cancer^57^. Compared to the corresponding normal tissues, the methylation level of WNT2/7B in tumor tissues was significantly reduced, leading to decreased transcriptional repression stability and subsequent overexpression. Conversely, WNT11 exhibited significantly increased methylation in tumor tissues, resulting in stable transcriptional repression, reduced expression, and potential impairment of its tumor-suppressive function. Notably, genomic alterations of key WNTs revealed that WNT11 had the most changes (5%), with amplification being the most common. This is an unusual association between the amplification and expression of tumor suppressor genes. Previous studies have shown that tumor suppressor amplification is rare and may signal genomic instability rather than active tumor suppression^58,59^. Further investigation is warranted to determine whether WNT11 amplification represents a compensatory response or byproduct of genomic instability during tumor development.

WNT2 and WNT11, which are positively correlated with immune scores in BRCA, are key players in TME modulation and may affect tumor progression and immune evasion. Immune cell correlation analysis revealed that WNT2 and WNT7B were immunosuppressive drivers, whereas WNT11 was an immune activator. These findings suggest that WNTs are dual modulators of BRCA immunity, and are correlated with immune cell infiltration and immunological functions in the TME. Moreover, positive correlations between WNTs and immune-related genes play a critical role in shaping the immune landscape in BRCA, influencing both immune activation and suppression. The association between WNT expression and chemokines suggests that WNT signaling may regulate immune cell trafficking and positioning within tumors. Single-cell RNA sequencing analysis from the TISCH2 database further confirmed the high expression of WNT genes in multiple immune cell subpopulations, including monocytes/macrophages, epithelial cells, malignant cells, myofibroblasts, endothelial cells, fibroblasts, and pericytes, underscoring their potential influence on immune modulation. Macrophages in the TME can polarize into two distinct phenotypes: M1 (pro-inflammatory and anti-tumorigenic) and M2 (immunosuppressive and tumor-promoting). M2 tumor-associated macrophages (TAMs) promote immune evasion by secreting immunosuppressive cytokines such as IL-10 and TGF-β, which inhibit T-cell activation and promote regulatory T cells (Tregs). Shifting macrophage polarization from M2 to M1 can enhance cytotoxic T-cell responses and improve the efficacy of immune checkpoint inhibitors (ICIs), facilitating better immune responses^60,61^. Tumor epithelial cells often undergo epithelial-to-mesenchymal transition (EMT), which reduces immune recognition and increases the metastatic potential. EMT downregulates MHC-I expression, rendering tumor cells less visible to cytotoxic T cells, and promoting resistance to apoptosis. Inhibition of EMT can restore immune cell recognition and improve the efficacy of immunotherapies by preventing tumor cell dissemination and enhancing T cell infiltration^62,63^. Malignant cells evade immune surveillance by upregulating immune checkpoint ligands such as PD-L1, which bind to PD-1 on T cells, leading to T-cell exhaustion and impaired anti-tumor responses. These cells also produce immunosuppressive cytokines such as TGF-β. Combining immune checkpoint inhibitors (anti-PD-1/PD-L1) with WNT pathway inhibitors can potentially reverse immune suppression in malignant cells, thereby enhancing T cell-mediated tumor clearance^64–66^. Endothelial cells contribute to angiogenesis and regulate immune cell trafficking. Aberrant endothelial cell function can create a blood-tumor barrier that limits immune cell infiltration into tumors^67,68^. Fibroblasts in the TME secrete cytokines and extracellular matrix proteins that support tumor growth and suppress immune function by creating a physical barrier^69,70^. Pericytes help stabilize tumor vasculature; however, their presence can contribute to the formation of dense blood vessels, limiting immune cell entry into the tumor^71^. Targeting WNT pathways may be a promising strategy for enhancing anti-tumor immunity and overcoming immune evasion mechanisms in BRCA.

The differential drug sensitivity of WNT2, WNT7B, and WNT11 suggests that these genes may serve as predictive biomarkers for therapeutic responses in breast cancer. The negative correlation between WNT2 and HDAC inhibitors (AR-42) and BET inhibitors (I-BET-762) implies that WNT2-expressing BRCA cells may resist epigenetic therapies, warranting combination therapeutic approaches. The negative correlation between WNT7B and Lapatinib and Afatinib (HER2-targeted therapies) suggests that WNT7B-overexpressing BRCA tumors may resist HER2-targeted therapies. Potential mechanisms underlying this resistance include: (1) β-catenin Signaling and HER2 crosstalk^72^(2) PI3K/AKT and MAPK/ERK activation^73^and (3) EMT induction^74^. Given these findings, targeting WNT7B-driven pathways in combination with HER2 inhibitors may be a potential strategy for overcoming resistance in patients with HER2-positive BRCA. The positive correlation between WNT11 and Piperlongumine, an oxidative stress inducer, suggests that targeting redox balance in BRCA cells overexpressing WNT11 may be a promising therapeutic strategy. The positive correlation between WNT11 and Simvastatin, and fluvastatin suggests that these lipophilic statins may play a role in targeting WNT11-driven breast cancer. Statins are known to disrupt lipid metabolism and mevalonate pathways, which are crucial for WNT signaling, making them potential adjuvant therapies for WNT-driven cancers^75,76^.

The enrichment of WNT2, WNT7B, and WNT11 proteins in pathways including “Wnt signaling”^7,50^“Hippo signaling”^77^“mTOR signaling pathway”^78^ and “PCP/CE pathways”^79^ underscores their central role in tumorigenesis. These pathways regulate cell proliferation, differentiation, migration and apoptosis. Dysregulation of these pathways can lead to uncontrolled cellular growth and metastasis, as observed in various cancers^50,77–79^. The identification of “Alzheimer’s disease”^80^ and “Pathways of neurodegeneration”^81^ in KEGG analysis is intriguing, as it suggests potential shared mechanisms between neurodegeneration and tumorigenesis, which could provide novel insights into the dual modulation of the Wnt pathway for therapeutic purposes. Enrichment of “frizzled binding” in GO analysis highlights the role of frizzled receptors, which are critical mediators of WNT signaling. Targeting frizzled receptors has shown promise in preclinical models for inhibiting metastasis and angiogenesis^82^. The significant enrichment of genes associated with the “endocytic vesicle membrane” suggests a role in the intracellular trafficking of receptors and ligands. Dysregulation of endocytosis is often linked to drug resistance in cancers, as it can alter the internalization and degradation of therapeutic targets such as tyrosine kinase receptors^83,84^. Enrichment in “GPCR ligand binding” supports GPCR modulation to influence the TME, immune response, and angiogenesis^85,86^.

This study has several limitations. First, the RNA expression levels in the present study could not be verified using protein expression levels. Transcriptomic data (mRNA expression) do not always correlate with protein expression because of post-transcriptional modifications and microRNA (miRNA) regulation. Experimental techniques, such as western blotting, immunohistochemistry (IHC), mass spectrometry, and flow cytometry, are required to confirm protein-level changes. Second, although this study integrated GEO datasets to ensure robust and consistent findings, enhancing the reliability of the results, dataset-specific biases, and batch effects may still influence the results. Third, this study utilized breast cancer cell line data from DepMap, which provides high-throughput functional genomic data, enabling drug sensitivity analysis and gene-dependency mapping in a controlled environment. However, cell lines lack tumor microenvironment interactions, immune components, and heterogeneity in patient tumors, limiting their physiological relevance. Fourth, we performed single-cell analysis using TISCH2, which revealed cell type-specific expression and avoided bulk RNA-seq averaging effects. However, its limitations include the absence of spatial transcriptomic integration, which may not fully represent BRCA heterogeneity across all subtypes, and lack of functional validation. Further validation using direct in vitro and in vivo studies are required. These findings will establish a basis for future studies to investigate the molecular mechanisms of WNTs relevant to the development and progression of BRCA.

## Conclusions

In conclusion, this study highlights the pivotal role of the WNT gene family in breast cancer progression, immune modulation, and therapy response. Our findings suggest that WNT2 and WNT7B act as potential oncogenic drivers, whereas WNT11 may serve as a tumor suppressor. Dysregulation of these genes driven by genetic and epigenetic changes may influence treatment resistance, particularly in HER2-positive BRCA. Surprisingly, elevated WNT2 expression is associated with improved survival outcomes, suggesting a paradoxical protective role. The association of WNT signaling with immune suppression and macrophage polarization highlights its relevance in immunotherapy, especially in combination with anti-PD-1/PD-L1 agents. Additionally, KEGG analysis revealed the enrichment of neurodegenerative pathways, suggesting potential molecular parallels between tumorigenesis and neurodegeneration. Future research should focus on integrating proteomic validation, spatial transcriptomics, preclinical drug testing, and functional assays to fully elucidate the mechanistic underpinnings of WNT signaling and its therapeutic implications in breast cancer. These findings lay the foundation for the development of targeted, WNT-modulating therapies for BRCA.

## Supplementary Information

Below is the link to the electronic supplementary material.

Supplementary Material 1.

Supplementary Material 2.

Supplementary Material 3.

Supplementary Material 4.

## Data Availability

The datasets we generated and/or analyzed during the current study are freely available in The Cancer Genome Atlas (TCGA) database (https://www.cancer.gov/tcga), UCSC XENA (https://xenabrowser.net/), GEPIA2 database (http://gepia2.cancer-pku.cn), Timer 2.0 database (http://timer.comp-genomics.org), UALCAN (http://ualcan.path.uab.edu/), Kaplan-Meier Plotter database (https://kmplot.com/analysis/), DepMap (https://depmap.org/), Shiny Methylation Analysis Resource Tool (SMART) App (http://www.bioinfo-zs.com/smartapp/), cBioPortal web database (https://www.cbioportal.org/), STRING database (https://string-db.org/), GDSC database (https://www.cancerrxgene.org/), and CTRP database (https://clinicaltrialsapi.cancer.gov/). The expression profile datasets GSE233242 and GSE227679 are available from the NCBI GEO database (https://www.ncbi.nlm.nih.gov/geo/). All data produced within this manuscript were attached as ‘Supplementary Materials’ file. The scripts used to perform the analysis in this are available in the following GitHub repository: https://github.com/bigbiolab/WNT_BRCA.
