# Supplementary material for: Comprehensive bioinformatics analysis reveals prognostic significance and immunological roles of WNT gene family in breast cancer
Source: Sci Rep. 2025 Oct 3;15:34490. doi: 10.1038/s41598-025-13315-6 (PMC12494856; doi:10.1038/s41598-025-13315-6)
Supplement: Supplementary file 1 — Supplementary Material 1. [file 41598_2025_13315_MOESM1_ESM.pdf]

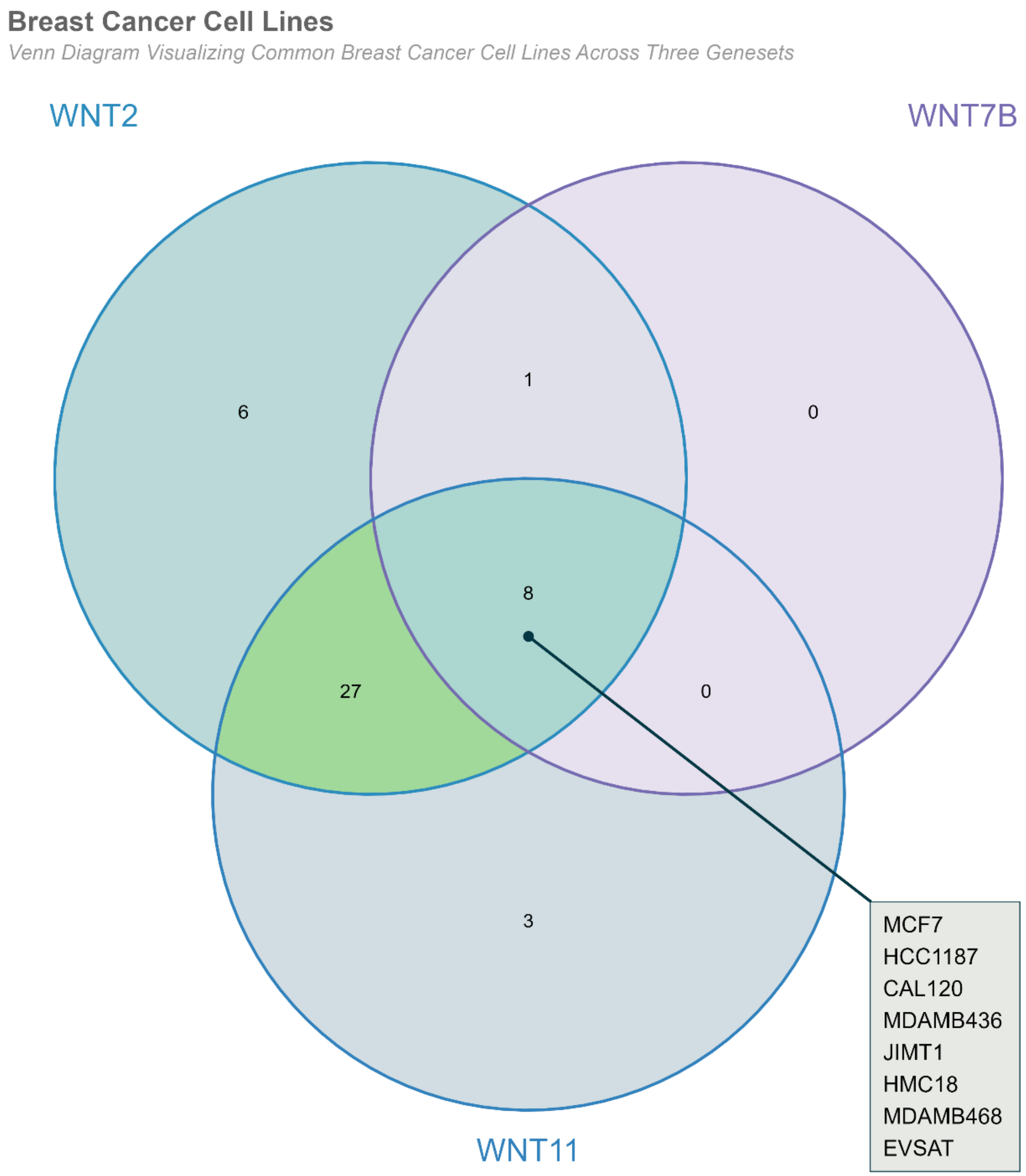

Supplementary Figure 1. Visualization of common patterns of negative gene effect score in breast cancer cell lines among WNT2, WNT7B, and WNT11.

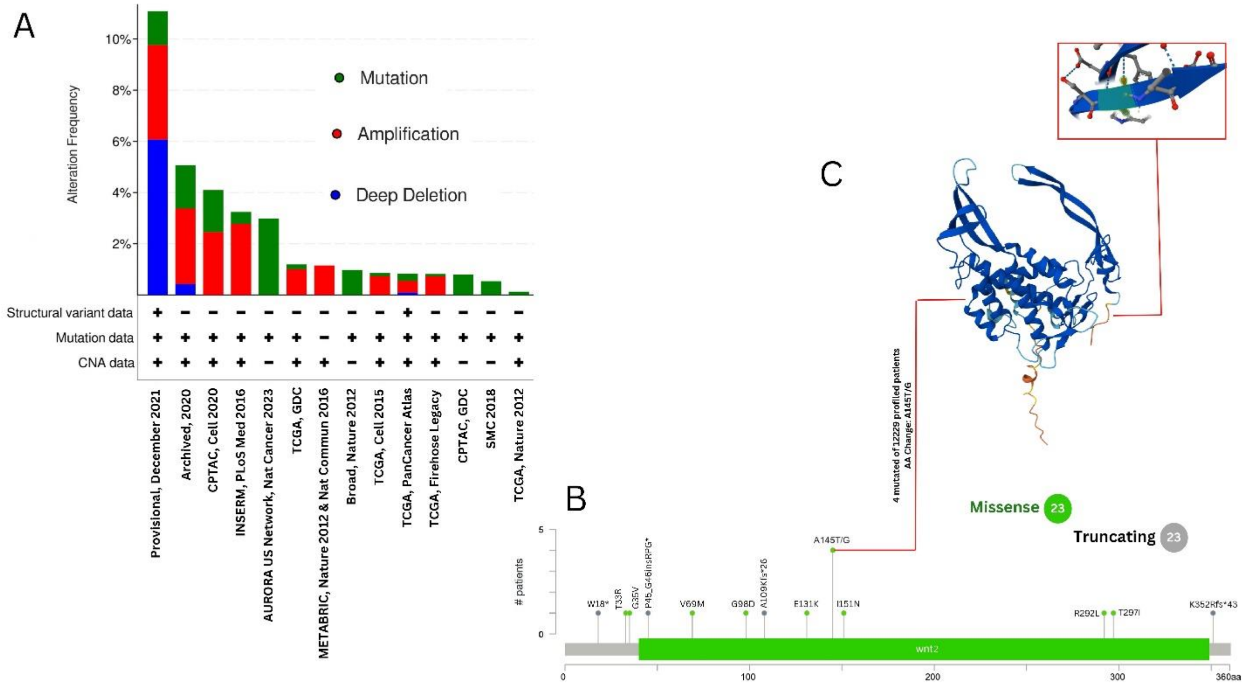

Supplementary Figure 2. The genetic alterations of WNT2 across BRCA cohort analysed by the cBioPortal database. (A) Alterations summary of WNT2 (B) The mutation types, number, and sites of the WNT2 genetic alterations. (C) 3D protein structure of WNT2 from AlphaFold.

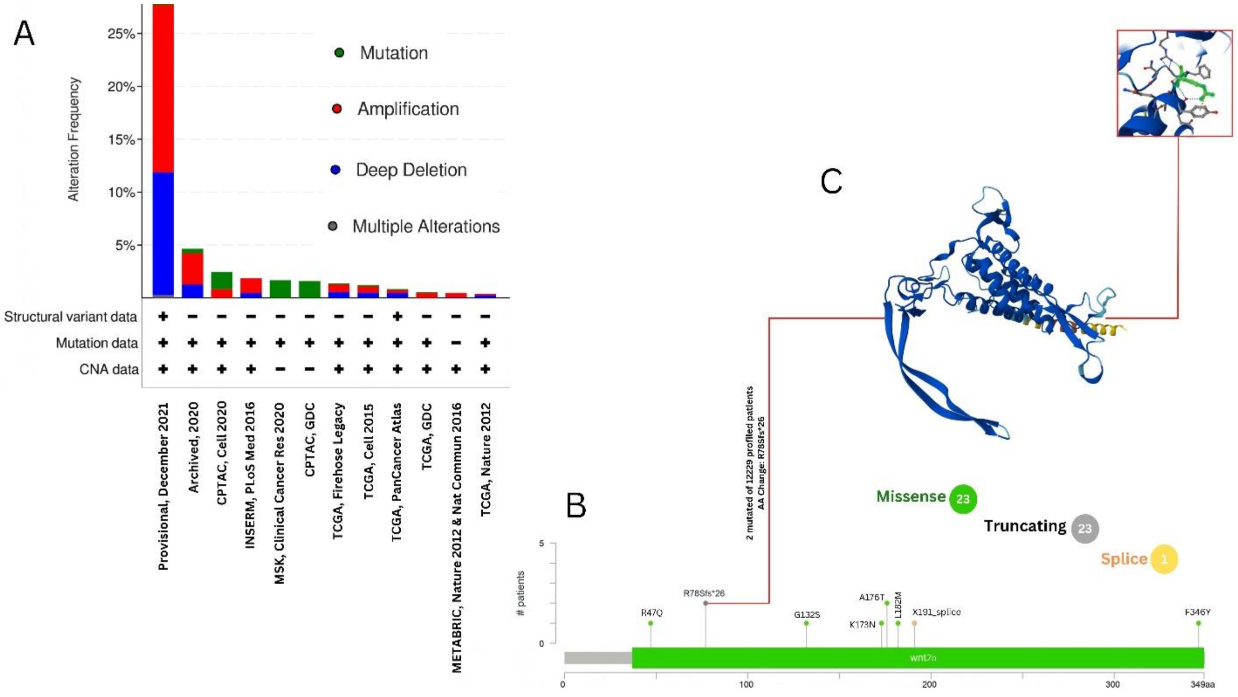

Supplementary Figure 3. The genetic alterations of WNT7B across BRCA cohort analysed by the cBioPortal database. (A) Alterations summary of WNT7B (B) The mutation types, number, and sites of the WNT7B genetic alterations. (C) 3D protein structure of WNT7B from AlphaFold.

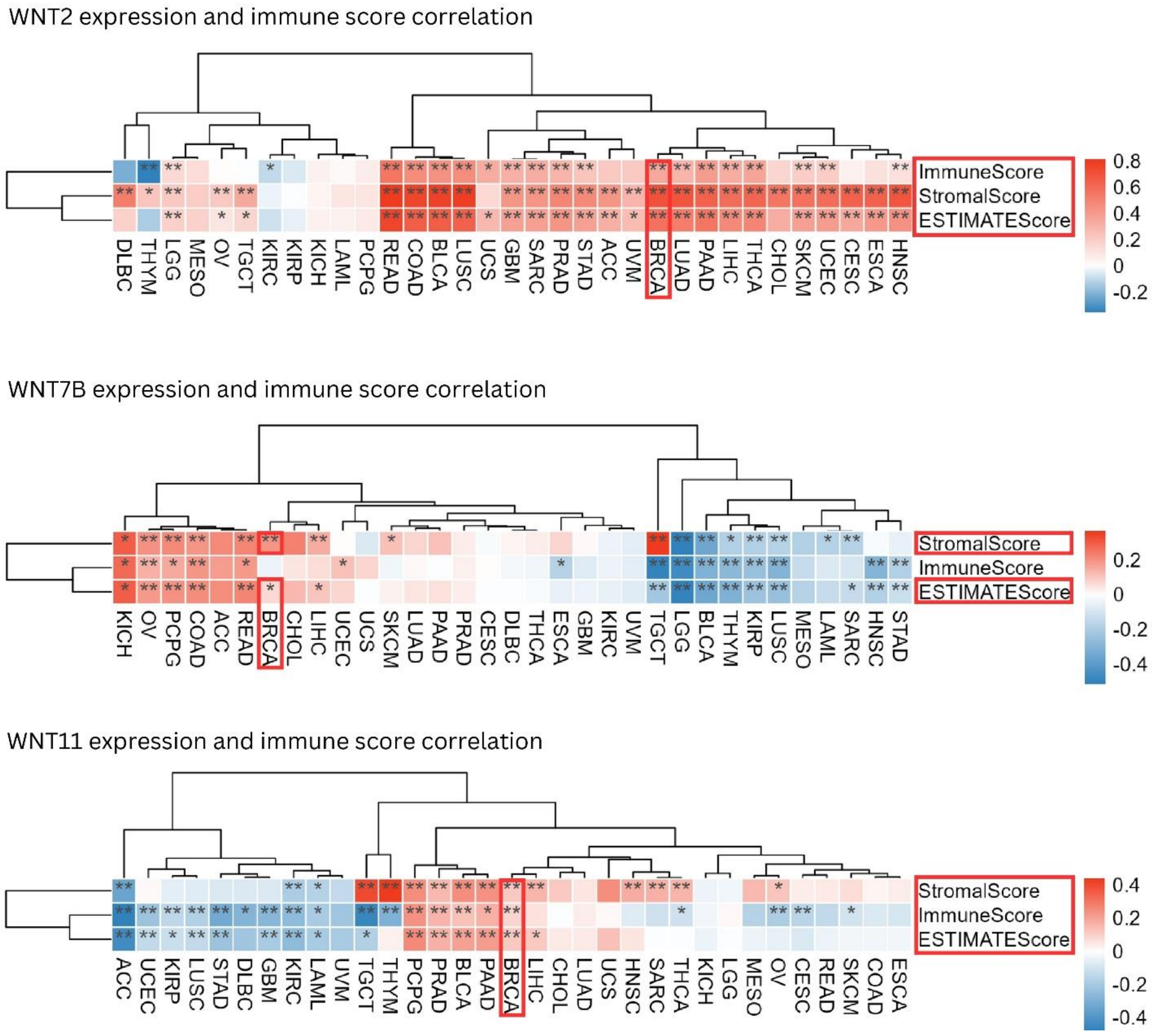

Supplementary Figure 4. Correlation analysis between WNT2, WNT7B, and WNT11 and immune scores. *P < 0.05, **P < 0.01.

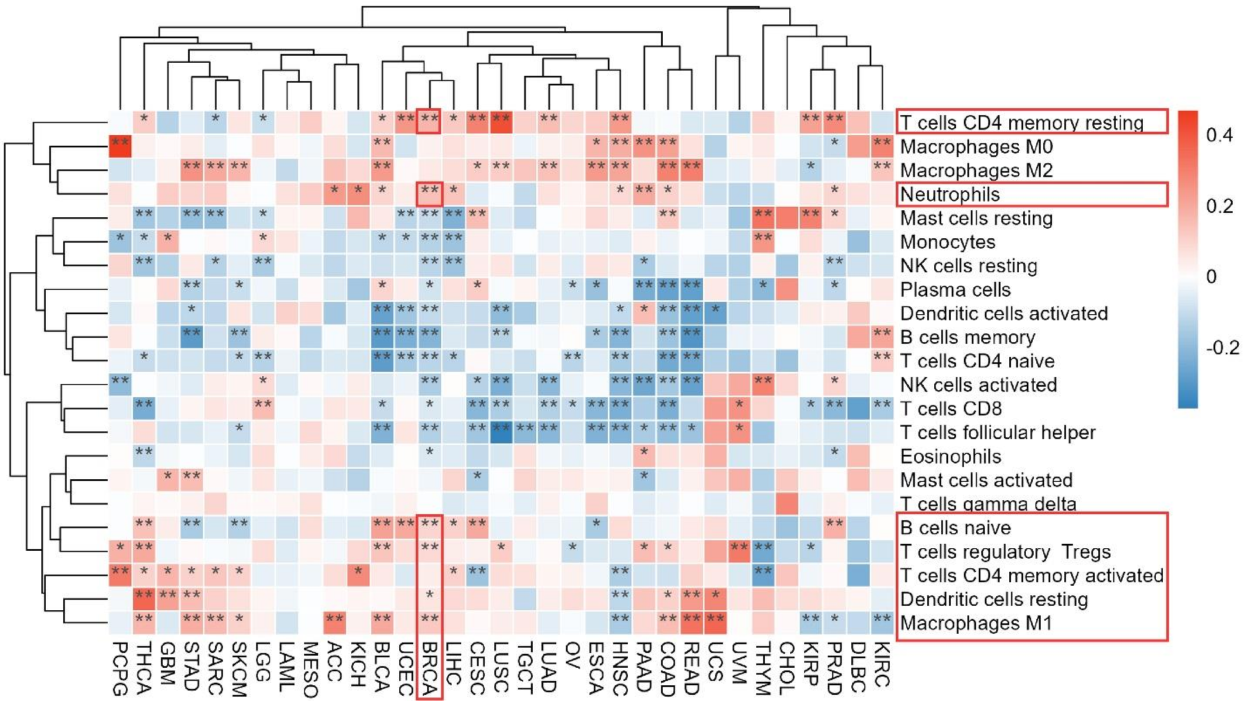

Supplementary Figure 5. The Pearson correlation analysis was used to calculate the correlation between WNT2 expression and immune cell ratio. *P < 0.05, **P < 0.01.

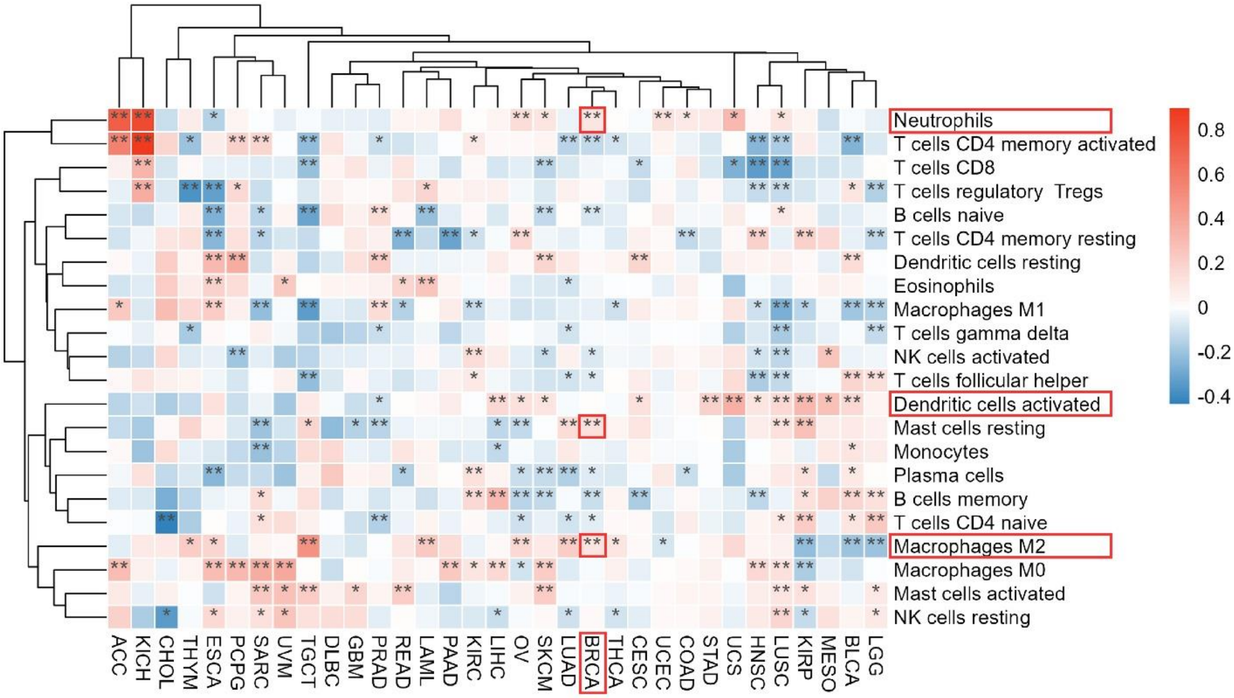

Supplementary Figure 6. The Pearson correlation analysis was used to calculate the correlation between WNT7B expression and immune cell ratio. *P < 0.05, **P < 0.01.

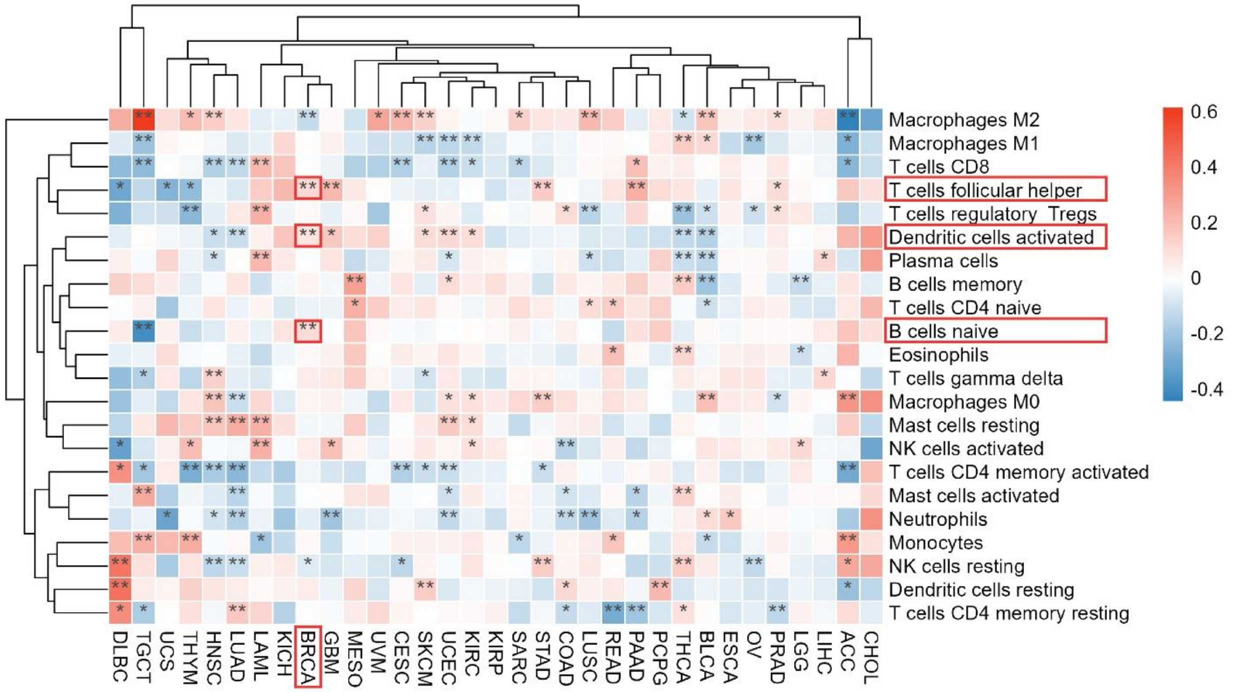

Supplementary Figure 7. The Pearson correlation analysis was used to calculate the correlation between WNT11 expression and immune cell ratio. *P < 0.05, **P < 0.01.

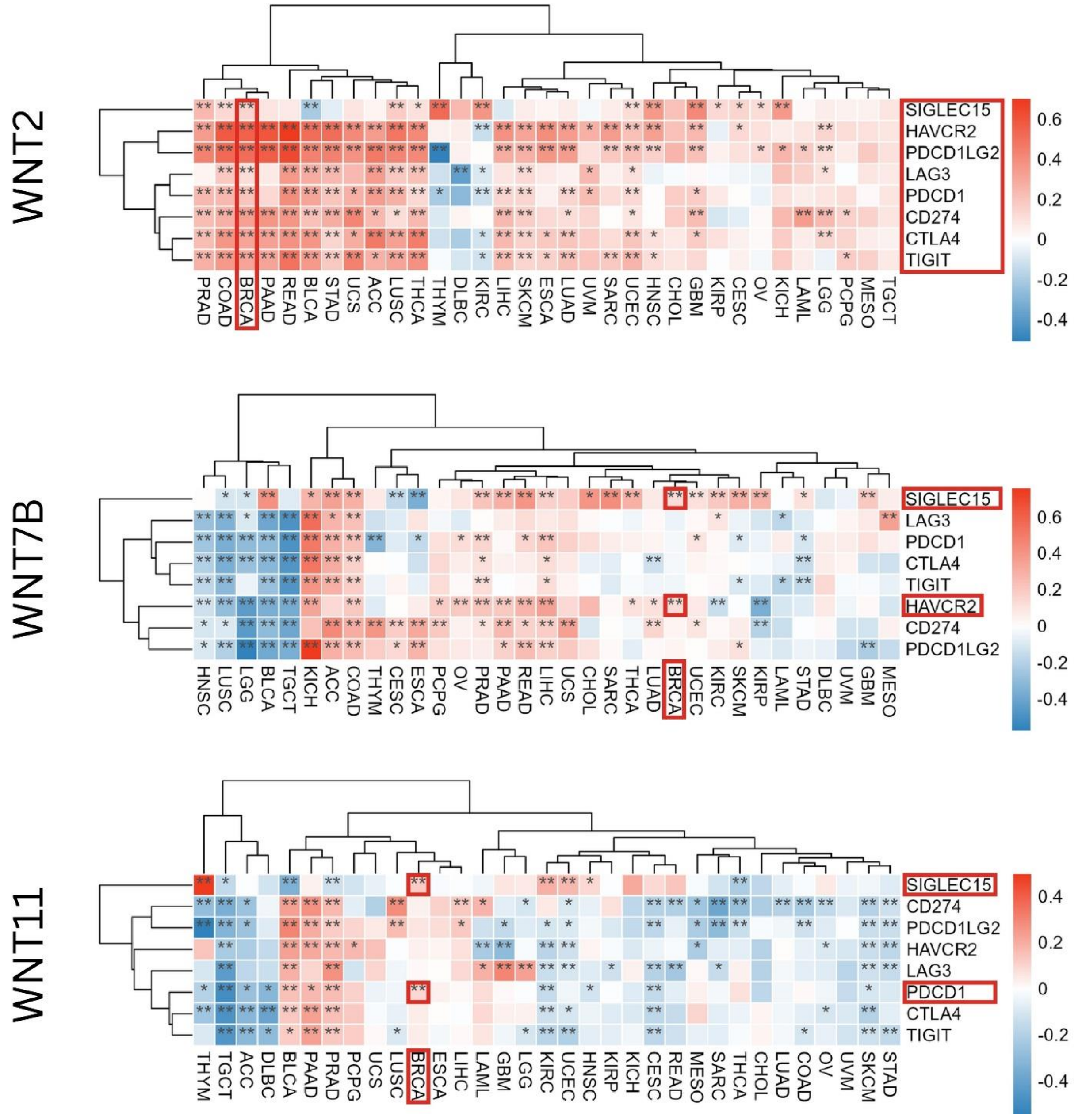

Supplementary Figure 8. Correlation between the expression of WNT2, WNT7B, and WNT11 with immune check point associated genes. *P < 0.05, **P < 0.01.

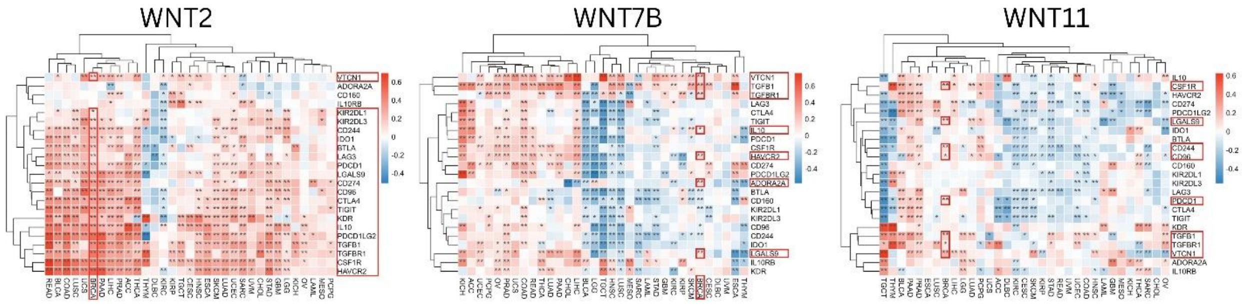

Supplementary Figure 9. Correlation between the expression of WNT2, WNT7B, and WNT11 with immune inhibitory genes. *P < 0.05, **P < 0.01.

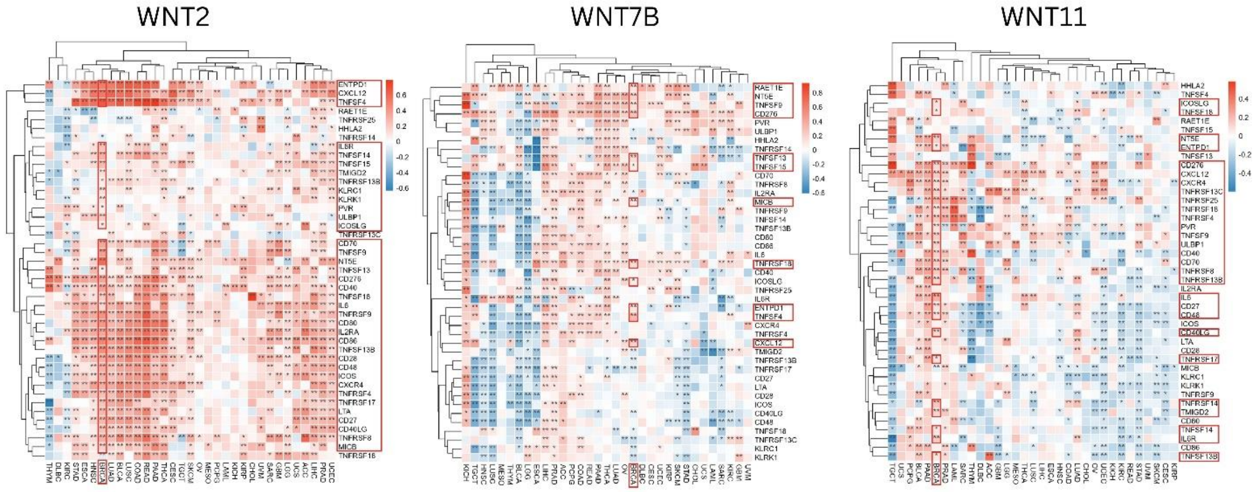

Supplementary Figure 10. Correlation between the expression of WNT2, WNT7B, and WNT11 with immune stimulating genes. *P < 0.05, **P < 0.01.

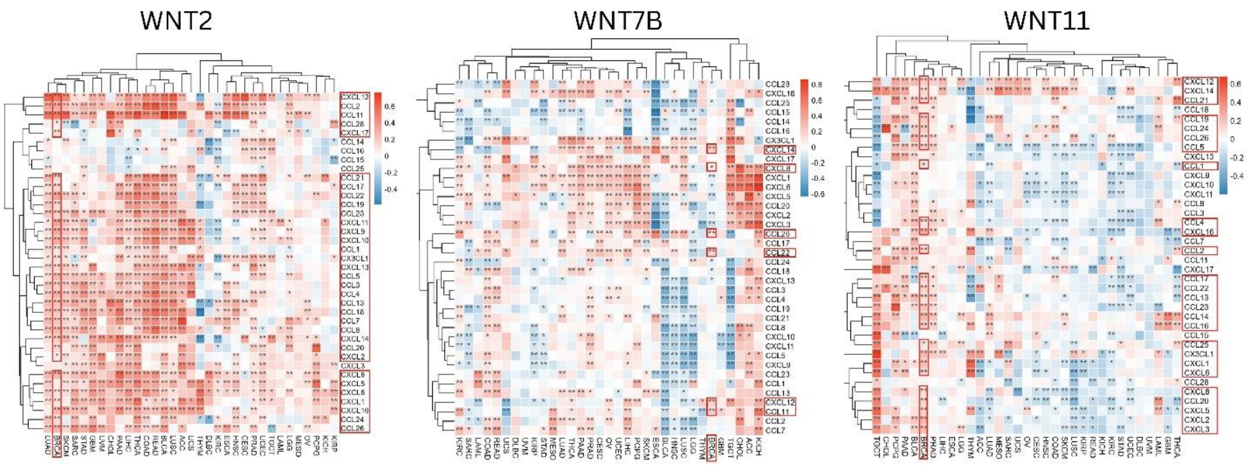

Supplementary Figure 11. Correlation between the expression of WNT2, WNT7B, and WNT11 with chemokines. *P < 0.05, **P < 0.01.

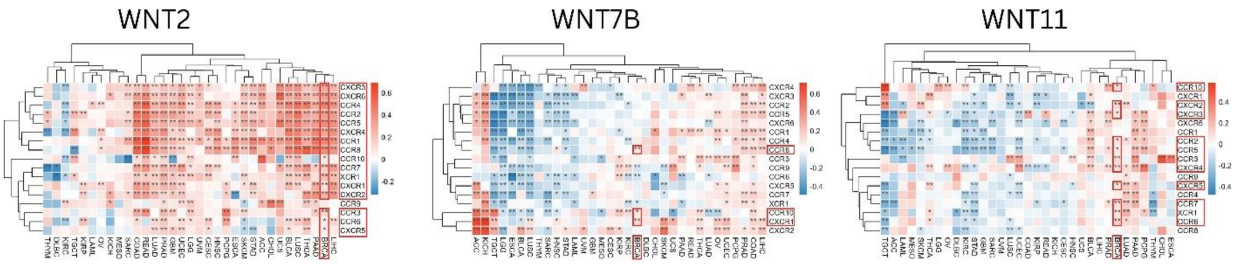

Supplementary Figure 12. Correlation between the expression of WNT2, WNT7B, and WNT11 with chemokine receptors. *P < 0.05, **P < 0.01.

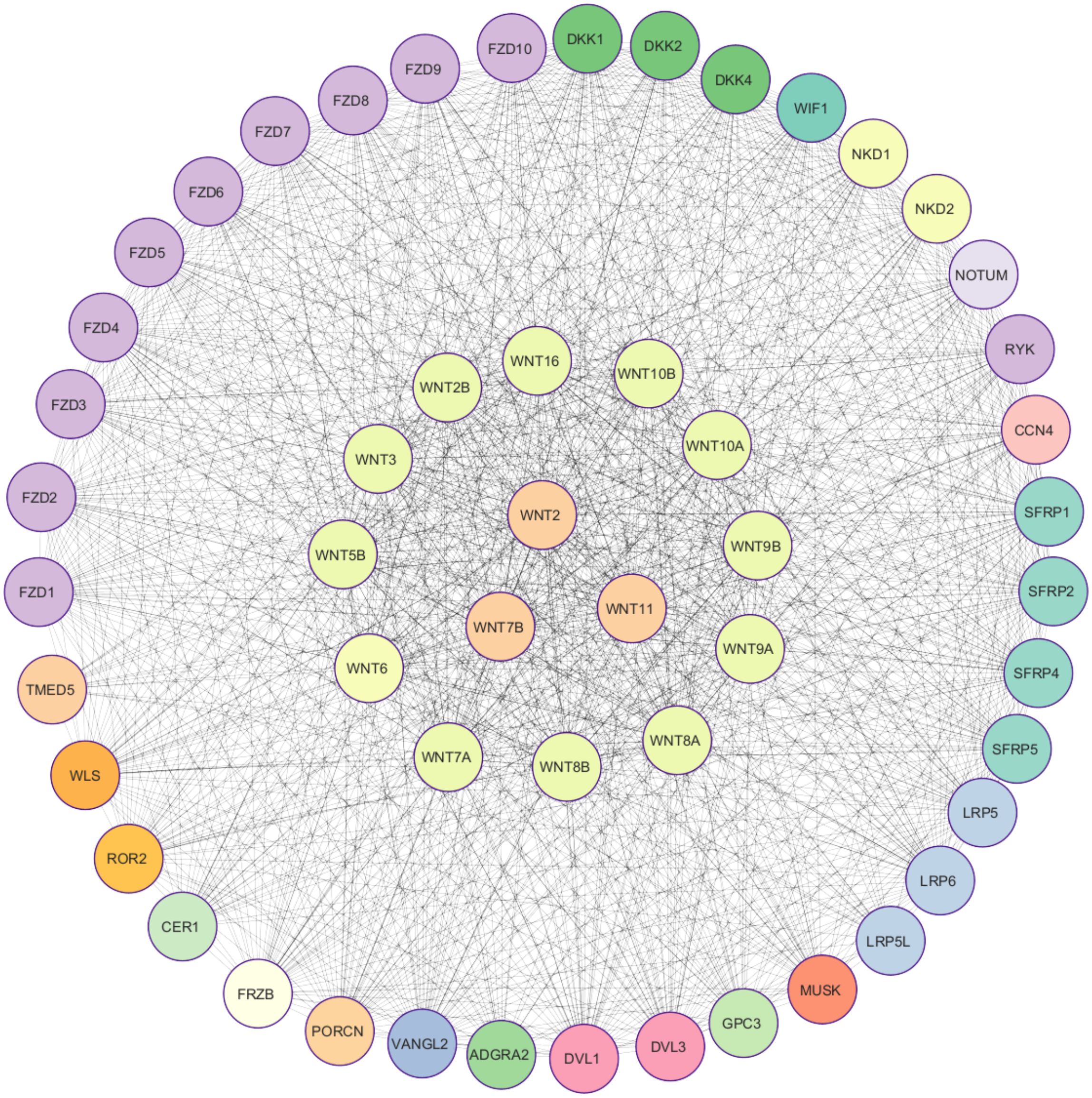

Supplementary Figure 13. The protein interaction network of 53 nodes and 1234 edges. These 52 genes were obtained from STRING tool, and the protein interaction network was modified by Cytoscape 3.9.1 software.
